# Enhanced climbing image nudged elastic band method with Hessian eigenmode alignment

**DOI:** 10.3389/fchem.2026.1807063

**Published:** 2026-05-29

**Authors:** Rohit Goswami, Miha Gunde, Hannes Jónsson

**Affiliations:** 1 Institute IMX and Lab-COSMO, École Polytechnique Fédérale de Lausanne (EPFL), Lausanne, Switzerland; 2 Science Institute and Faculty of Physical Science, University of Iceland, Reykjavík, Iceland; 3 Division of Theoretical Physics, Institute Ruđ er Bošković, Zagreb, Croatia

**Keywords:** Bayesian regression, high-throughput chemistry, hybrid optimization, machine learning potentials, nudged elastic band, potential energy surface, reaction kinetics, transition state search

## Abstract

Accurate determination of the transition states is central to an understanding of reaction kinetics. Double-endpoint methods where both the initial and final states are specified, such as the climbing image nudged elastic band (CI-NEB), identify the minimum energy path between the two and thereby the saddle point on the energy surface that is relevant for the given transition, thus providing an estimate of the transition state within the harmonic transition state theory. Such calculations can, however, incur high computational costs and may suffer stagnation on exceptionally flat or rough energy surfaces. Conversely, methods that only require the specification of an initial set of atomic coordinates, such as the minimum mode following (MMF) method, offer efficiency but can converge on saddle points that are not relevant for the transition of interest. Here, we present an adaptive hybrid algorithm that switches between the CI-NEB and the MMF methods so as to achieve faster convergence to the relevant saddle point. The method is benchmarked On for the Baker–Chan (BC) saddle point test set using the PET-MAD machine-learned potential, along with 59 transitions of a heptamer island on Pt (111) from the OptBench set. A Bayesian analysis of the performance shows a median reduction of energy and force calculations by 57% [95% CrI: 64%, −50%] relative to CI-NEB for the BC set, while a 31% reduction is found for the transitions of the heptamer island. Calculations of the BC set, where a simple switch from the CI-NEB to the MMF method is made when the magnitude of the atomic forces decreases below 0.5 eV/Å, requires 46% more force calculations than the OCI-NEB algorithm. These results show that an adaptive hybrid method mixing CI-NEB and MMF can be a highly efficient tool for high-throughput automated chemical discovery of atomic rearrangements.

## 1 Introduction

Estimating reaction kinetics requires an accurate free energy barrier corresponding to the transition state that is a dividing surface that forms a bottleneck for reactive trajectories. Within the harmonic approximation to transition state theory (HTST), the transition state is approximated as a 3N−1 dimensional hyperplane between the reactant and product basins of the potential energy surface (PES). The plane passes through a first-order saddle point, which we will refer to as the transition structure (TS), and its normal points in the direction of lowest curvature of the PES at that point. The relevant point is the point of highest energy along the minimum energy path (MEP) connecting the reactant and the product states, and this is a saddle point on the PES. For a given reaction, the MEP is the path of the highest statistical weight in the configuration space connecting the initial and final states.

TS exploration methods identify a candidate transition mechanism. The estimate of the transition rate (under HTST) can be calculated from the energy barrier, which is the difference between the energy at the saddle and the reactant state.

According to the *a-priori* knowledge of the system, TS exploration methods are classified as “double-endpoint” and “initial-point” methods. The former assumes knowledge of two sets of atom coordinates corresponding to the local energy minima, representing the reactant and product states, and finds an MEP between the two. This includes the nudged elastic band or NEB (Jonsson et al. 1998), where a discrete chain of images or configurations of atoms connecting the known reactants and products relax to the MEP. By construction, the NEB method defines a discrete representation of a path in the configuration space, and the calculation requires the computation of energy and atomic forces for each discrete configuration along the path. If this requires electronic structure calculations, the computational effort can become large. A key issue in a successful NEB calculation is the choice of the initial path. In addition, the choice of optimizers strongly affects the convergence rate and efficiency. Estimating the saddle point geometry from the results of an NEB calculation relies on having configurations in its vicinity. In turn, this is controlled by the number of images along the path and the spring constant, which determines their distribution along the path. To converge onto the saddle point, one of the images, the climbing image, can be made to move uphill along the path (CI-NEB) (Henkelman et al., 2000). NEB calculations may require many iterations for flat or rough potential surfaces where the atomic forces are small or vary sharply between the adjacent images.

Conversely, the “initial-point” methods require only a single starting configuration of the atoms from which the exploration for the TS begins. These methods, such as the dimer Henkelman and Jónsson (1999) method, typically follow the lowest eigenmode of the Hessian (i.e., curvature of the PES) to a saddle point. Such minimum-mode following (MMF) searches do not require the evaluation of multiple configurations for each traversal step. Due to the unconstrained nature of the search, a saddle point may be found that is irrelevant for the reaction of interest, or an already known saddle point is rediscovered, which offsets the computational efficiency.

Herein, we describe an off-path climbing image nudged elastic bands (OCI-NEB), a method integrating the stability of a double-endpoint chain-of-replica method with the efficiency of an MMF saddle point search method. While a switch from a double-endpoint method when a certain level of convergence is obtained to an initial-point method to complete the search has previously been practiced by Ásgeirsson et al. (2021) and Park H. et al. (2025), the OCI-NEB can switch between the two, back and forth, depending on certain properties that are monitored during the calculation. We show that by coupling this adaptive triggering with an alignment-based cool-down strategy, OCI-NEB uniformly accelerates convergence, more than halving the number of energy and atomic force evaluations for two dissimilar benchmark sets.

## 2 Methods

Before presenting the proposed OCI-NEB algorithm, the dimer and the nudged elastic band methods are reviewed for completeness based on the implementation in the eOn^1^ software suite (Goswami, 2025b).

### 2.1 Minimum mode following: the dimer method

To locate the first-order saddle points, in principle, the full Hessian matrix at each point is required. The Hessian is a 3N x 3N matrix, with each element requiring at least one force call, which becomes computationally prohibitive for most systems. To work around calculating the full Hessian, the minimum mode following algorithms assume that the most relevant mode is the eigenvector corresponding to the lowest eigenvalue, the “minimum mode.” The minimum mode can be computed approximately without the full Hessian (Mousseau et al., 2012; Munro and Wales, 1999) following the eigenvector approach of Cerjan and Miller (1981). In this work, the minimum mode is found at each step using the dimer method (Henkelman and Jónsson, 1999; Olsen et al., 2004).

The “dimer” in the dimer method consists of two replicas, namely, $R_{1}$ and $R_{2}$ , which are displaced symmetrically from a central point R by a small half-separation ΔR/2 along a normalized orientation vector $\hat{N}$ as in Equation 1:

$$R_{1}=R-\frac{\Delta R}{2}\hat{N},\;R_{2}=R+\frac{\Delta R}{2}\hat{N}.$$ (1)

This dimer construct undergoes constrained relaxation, or “rotation,” such that the separation ΔR does not change and the mid-point remains fixed. This procedure brings $\hat{N}$ (the dimer axis) closer to the lowest curvature mode at each step. The final axis after the “rotation” is indicated by $\hat{d}$ . The dimer axis is the approximation to the lowest curvature mode of the Hessian. The curvature C along the dimer axis is approximated by a central finite difference of the forces $F_{1,2}=-\nabla V\left(R_{1,2}\right)$ acting on the endpoints. With the convention above ( $R_{1}$ is the “minus” endpoint), the curvature is found by the Rayleigh-quotient estimate and is depicted as (Equation 2) (Henkelman and Jónsson, 1999):

$$C\left(\hat{N}\right)={\hat{N}}^{T}H\hat{N}\approx \frac{\left(F_{1}-F_{2}\right)\cdot \hat{N}}{\Delta R}.$$ (2)

Once aligned, the central configuration R translates under a modified effective force $F_{\text{trans}}$ obtained by inverting the component of the true force along the dimer axis $\hat{N}$ . This modification converts what would be gradient ascent along $\hat{N}$ into effective descent, while leaving the force components along all other directions unchanged. This inversion of the force along a component can also be understood as a Householder transformation along the vector N , which can be written as $T_{H}=I-2\hat{N}\cdot {\hat{N}}^{T}$ . The translation then becomes (Equation 3)

$$\begin{array}{lll} F_{\text{trans}}\left(R\right) & =F\left(R\right)-2\left(F\left(R\right)\cdot \hat{N}\right)\hat{N} & =T_{H}F. \end{array}$$ (3)

The dimer rotation and translation steps alternate until the force norm drops below a convergence threshold, at which point a saddle point has been located.

The MMF method may be started from any point on the energy surface. It can find a collection of saddles representing the possible transitions from a given initial state by starting from the displaced configurations near the minimum. Alternatively, the MMF can refine a guess obtained by other means, and we utilize this concept in the present work to iteratively improve a partially converged double-end-point calculation.

### 2.2 Nudged elastic band—NEB

Double-endpoint searches are often used to find the full MEP between the known reactant and product states. We used the nudged elastic band method, which approximates the reaction pathway as a chain of “images” connected by fictitious springs. The force acting on each image is

$$F_{i}^{\text{NEB}}=F_{i}^{⊥}+F_{i}^{\|,\text{spring}}.$$ (4)

The method ensures convergence to the MEP and controls the distribution of the images along the path by projecting the force components according to an estimate of the local tangent to the path. The true potential force only acts perpendicular to the path tangent,

$$F_{i}^{⊥}=F_{i}^{\text{true}}-\left(F_{i}^{\text{true}}\cdot {\hat{\tau }}_{i}\right){\hat{\tau }}_{i},$$ (5)

while the spring forces act only parallel to it.

$$F_{i}^{\|,\text{spring}}=k\left(\left|R_{i+1}-R_{i}|-|R_{i}-R_{i-1}\right|\right){\hat{\tau }}_{i}.$$ (6)

This force projection—referred to as “nudging”—decouples the optimization of the path-shape from the distribution of images along the path.

For improved numerical stability, the “improved tangent” estimate is used (Henkelman and Jónsson, 2000), which defines the tangent based on the neighboring image that is higher in energy to reduce the probability of kinks in the path,

$${\hat{\tau }}_{i}=\left\{\begin{array}{cc} \text{normalize}\left(R_{i+1}-R_{i}\right) & \text{if }V_{i+1}>V_{i}>V_{i-1}\\ \text{normalize}\left(R_{i}-R_{i-1}\right) & \text{if }V_{i-1}>V_{i}>V_{i+1}\\ \text{weighted average} & \text{otherwise}. \end{array}\right.$$ (7)

At extrema, the tangent is defined to be the weighted average of the vectors to neighboring images, with preference given to the vector on the higher energy side.

To converge rigorously on the saddle point, the highest-energy image is turned into a “climbing image” (Henkelman et al., 2000), where the spring force is removed, and the parallel component is inverted. This forces the selected image, identified as the “climbing” image, to move uphill along the path to the saddle point.

$$F_{\text{climb}}=F_{\text{climb}}^{\text{true}}-2\left(F_{\text{climb}}^{\text{true}}\cdot {\hat{\tau }}_{\text{climb}}\right){\hat{\tau }}_{\text{climb}}.$$ (8)

The accuracy of the saddle configuration for the CI-NEB calculations depends on the tangent approximation, which, in turn, derives from the neighboring images of the climbing image. To increase the resolution of the images around the saddle without increasing the number of images, the springs can be adjusted either through geometric considerations (Ásgeirsson et al., 2021) or kinetic considerations (Mandelli and Parrinello, 2021).

The energy-weighted spring method (Ásgeirsson et al., 2021) used here dynamically stiffens the spring constant in high-energy regions, thus concentrating the chain to enforce higher image density near the saddle point.

The quality of the initial path influences the convergence speed and stability for the NEB. Linear interpolations on atomic positions often produce unphysical atomic overlaps, particularly for complex or dense systems. To mitigate this without having to construct new paths for every system, improved initial paths in Cartesian coordinates can be constructed using the sequential image dependent pair potential (S-IDPP) method (Schmerwitz et al. 2024; Smidstrup et al., 2014).

The S-IDPP constructs the path by sequentially growing images from the reactant and product endpoints. It optimizes these intermediate images on a simplified auxiliary surface defined by interpolating the pairwise distances of bonded atoms at the endpoints. This procedure attempts to eliminate high-energy steric clashes before engaging the computationally intensive electronic structure or MLIP calculations.

### 2.3 Off-path climbing image nudged elastic band (OCI-NEB)

The standard climbing image nudged elastic band, or CI-NEB, depends on the efficiency of the underlying optimizer. On flat or noisy sections of the PES, the projected force components can oscillate or become ill-defined. To address this, we introduce the OCI-NEB algorithm, which is a hybrid approach that intersperses standard CI-NEB optimization steps with targeted MMF refinement using the dimer method as implemented in eOn (see https://eondocs.org) (Heyden et al., 2005; Olsen et al., 2004).

This method builds on a two-stage refinement strategy (Goswami, 2025b) in which dimer calculations are carried out within NEB iterations once the path relaxes sufficiently. The NEB first brings the climbing image near the saddle point; a short burst of single-image MMF then refines the climbing image position before the NEB resumes.

In practice, a fixed number of steps of CI-NEB initially (Goswami, 2025b) or a single threshold for switching from CI-NEB to MMF does not transfer well across the benchmark systems and energy surfaces. Workflow methods (Park H. et al., 2025) or a fixed tolerance on the magnitude of the atomic forces for switching to MMF (Ásgeirsson et al., 2021) may not lead to the appropriate saddle connecting the given reactant and product basins.

OCI-NEB dynamically switches between the NEB and dimer during a single calculation rather than switching just once from CI-NEB to MMF. This is inspired by the active learning set of acceleration methods (Peterson, 2016; Koistinen et al., 2019; Koistinen et al., 2020; Goswami and Jónsson, 2025; Goswami et al., 2025a) and means establishing bidirectional coupling, which allows switching from the MMF back to the NEB based on a stability mechanism to ensure improvement. The OCI-NEB, therefore, ensures convergence in the region of interest. We develop the algorithm below by addressing the stability and efficiency challenges inherent to such hybridization.

#### 2.3.1 Approximations to the lowest mode: NEB and dimer

Equations 3, 8 share an identical algebraic structure; both are Householder reflections of the true force through unit vector $\hat{\chi }$ , $\hat{d}$ for the dimer, and $\tau_{\text{climb}}$ for the NEB,

$$F_{\text{eff}}\left(R,\hat{\chi }\right)=T_{H}F\left(R\right).$$ (9)

The reflector $T_{H}$ inverts the force component along $\hat{d}$ while leaving the orthogonal complement unchanged. This force inversion allows a standard minimization algorithm (L-BFGS, FIRE, and CG) to converge to a saddle point: the modified force $T_{H}F$ has a zero at the saddle $\left(T_{H}0=0\right)$ , and the inversion along the lowest mode enforces ascent along that direction (Weinan et al., 2007). Since both the climbing image and the dimer modify the dynamics to converge toward a saddle state on the energy surface, switching between the dimer and the NEB as in the OCI-NEB does not affect the state and does not meaningfully change the final solution.

**FIGURE 1:**
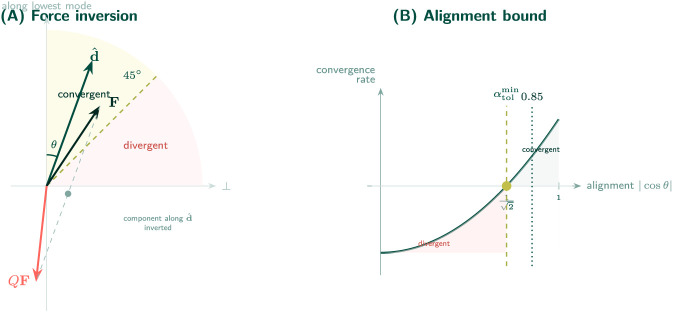
Force inversion geometry and the bound alignment. **(A)** The dimer axis $\hat{d}$ defines the direction along which the force is inverted. The reflector $T_{H}=I-2\hat{d}{\hat{d}}^{T}$ maps the force F to $T_{H}F$ (coral). When $\hat{d}$ lies within 45° of the lowest curvature mode, as approximated by the NEB (yellow cone), the modified force drives the configuration toward the saddle. Outside this cone (pink), the force inversion pushes away from it. **(B)** Critical alignment: the cosine of the angle θ between $\hat{d}$ and the NEB-axis τ must exceed $1/\sqrt{2}$ for convergent behavior. Below this threshold, the modified dynamics lead away from the saddle. The dashed line marks $\alpha_{\text{tol}}^{\min}=1/\sqrt{2}$ ; at the dotted line at 0.85, the empirical optimum from the data generated.

When the dimer moves the climbing image off-path, subsequent NEB iterations must redistribute images before the climbing image reactivates, thus incurring additional force evaluations. Alternately, if $\hat{d}$ is misaligned by more than 45° from the lowest mode, the inversion can push the configuration away from the saddle rather than toward it. This critical angle corresponds to the requirement $|\hat{d}\cdot {\hat{v}}_{\min}|>1/\sqrt{2}$ , where ${\hat{v}}_{\min}$ is the dimer’s estimate of the lowest mode (see Section 4 for the derivation). In OCI-NEB, the dimer is initialized along the NEB tangent $\hat{\tau }$ and then rotated; the alignment $\alpha =|{\hat{v}}_{\min}\cdot \hat{\tau }|$ measures how well the dimer mode matches the path direction. The requirement $\alpha \geq 1/\sqrt{2}$ ensures that the force inversion drives the climbing image toward the saddle of interest. Figure 1 illustrates this geometry.

We never need the full Hessian—only the dimer’s one-mode estimate.

#### 2.3.2 Dynamic control via mode alignment

A primary challenge for hybrid methods involves determining the duration of the refinement phase. OCI-NEB utilizes the alignment between the dimer axis $\hat{d}$ and the NEB path tangent $\hat{\tau }$ . By initializing the dimer along $\hat{\tau }$ , we ensure the dimer search begins in a relevant subspace, preventing initialization-based failures (Goswami and Jónsson, 2025). The alignment between the two parameters provides an early stopping criterion: the MMF continues while the orientation remains close to the tangent estimate. When the angle grows large, the MMF terminates and the optimizer history determines the climbing image position along the dimer trajectory with the most negative eigenvalue. This termination criterion replaces arbitrary step counts with a physically motivated bound.

$$\alpha =|\hat{d}\cdot \hat{\tau }|.$$ (10)

#### 2.3.3 Relative baselines and transferability

Given that we generate initial paths using S-IDPP, which may involve high-energy images, defining the “handover point” or the force threshold at which the algorithm switches from the NEB to MMF becomes problematic for high-throughput calculations. Defining absolute force thresholds, e.g., 0.5 eV/Å, does not work well across a diverse dataset of different reaction mechanisms, since a threshold value appropriate for stiff covalent bond breakage may correspond to an effectively converged state for a soft supramolecular rearrangement.

To mitigate this, we establish a relative baseline. At the start of the calculation, we record the baseline convergence force $\left(F_{0}\right)$ , which is the maximum force norm on any atom of the initial path. We then express the triggering threshold $T_{\mathrm{mmf}}$ relative to this baseline:

$$T_{\mathrm{mmf}}=\lambda_{\mathrm{trigger}}F_{0}.$$ (11)

The algorithm, therefore, engages only after the NEB has relaxed the high-energy configurations from the initial guess, regardless of the absolute energy scale of the system.

#### 2.3.4 Robustness: stability latches and restoration

Despite the early stopping criteria and the initial relative thresholds, activating the MMF based solely on the force criteria can lead to instability for rough potentials or where the climbing image index oscillates. The highest energy image along the path, which is selected for the climbing image, especially for automated initial paths, may oscillate during the initial phase of the calculation. The OCI-NEB implements a stability latch, switching to the dimer only if the climbing image index remains constant for κ consecutive iterations.

The internal state of the optimizer resets if the motion of the image exceeds the maximum move of each image times the total number of intermediate images. Two distinct restoration mechanisms protect against failed MMF searches. If the dimer encounters positive curvature (eigenvalue >0 ), indicating that the climbing image has drifted to a local minimum rather than a saddle, the image is restored to its pre-MMF position, and the cached eigenvector is discarded; the NEB then resumes from the unperturbed state. For all other termination modes (alignment failure and step budget exhaustion), the climbing image advances to the configuration with the most negative curvature found during the search. Both mechanisms allow OCI-NEB to recover partial progress from a terminated MMF search before returning control to the NEB.

#### 2.3.5 Failure recovery

When the dimer dephases from the NEB tangent, the triggering threshold must be raised to prevent repeated premature activation. We parameterize the penalty through a one-parameter family $P\left(\alpha ,S\right)=B+\left(1-B\right)\alpha^{S}$ with B=1/(1+S) , which is subject to the boundary conditions P(0)=B (maximum penalty at zero alignment) and P(1)=1 (no penalty at perfect alignment). Within this family, the unique affine member is selected by requiring $P^{\prime \prime }\equiv 0$ on (0,1). It is computed as:

$$\frac{\partial^{2}P}{\partial \alpha^{2}}=\frac{s^{2}\left(s-1\right)}{1+s}\;\alpha^{s-2},$$ (12)

which is identically absent on (0,1) if and only if s=1 , since $\alpha^{s-2}>0$ and $s^{2}/\left(1+s\right)>0$ for all the positive s . Setting S=1 gives B=1/2 , and the penalty reduces to

$$P\left(\alpha \right)=\frac{1}{2}+\frac{1}{2}\;\alpha .$$ (13)

Any S<1 yields a concave penalty (too aggressive near full alignment), while S>1 yields a convex penalty (too lenient near zero alignment). The linearity condition eliminates both failure modes. At zero alignment, the threshold halves; at perfect alignment, no penalty applies. The on-failure threshold then reads as

$$T_{\text{fail}}=F_{0}\;\lambda_{\text{rel}}\;P\left(\alpha \right),$$ (14)

so the penalty strength and shape are fully determined by the linearity constraint, requiring no additional tuning beyond $\lambda_{\text{rel}}$ .

#### 2.3.6 Post-MMF path reparameterization

When the dimer moves the climbing image, the neighboring NEB images become unevenly spaced along the path. Rather than relying on the spring forces to gradually redistribute the images over subsequent NEB iterations, we apply an arc-length reparameterization immediately after each successful MMF step (i.e., when $F_{\text{new}}<F_{\text{CI}}$ ). This redistributes all the intermediate images at equal arc-length intervals via cubic Hermite interpolation using the same procedure as the S-IDPP initialization. The reparameterization is geometry-only (zero additional force evaluations); the forces are recomputed in the next NEB iteration regardless. The L-BFGS optimizer state is reset after reparameterization to prevent stale gradient history from corrupting the search direction.

#### 2.3.7 Success threshold update

When the MMF step reduces the climbing image force $\left(F_{\text{new}}<F_{\text{CI}}\right)$ , the triggering threshold is updated to permit re-entry at a level that is proportional to the achieved improvement:

$$T_{\text{success}}=F_{\text{new}}\left(\frac{1}{2}+\frac{2}{5}\;\frac{F_{\text{new}}}{F_{\text{CI}}}\right).$$ (15)

The two limiting cases motivate the form, namely, when $F_{\text{new}}\approx F_{\text{CI}}$ (marginal improvement), $T_{\text{success}}\approx 0.9\;F_{\text{CI}}$ , setting the threshold just below the current force to require further NEB relaxation before re-triggering. When $F_{\text{new}}\ll F_{\text{CI}}$ (substantial improvement), $T_{\text{success}}\approx 0.5\;F_{\text{new}}$ , which encourages early re-entry into the MMF phase. At convergence $\left(F_{\text{new}}\to 0\right)$ , the threshold is absent and the NEB takes over.

#### 2.3.8 Complete algorithm

The complete OCI-NEB procedure is given in Algorithm 1.

Algorithm 1 **Off-path climbing image nudged elastic band (OCI-NEB).**

1: **Input:** Initial Path $P=\left\{R_{0},\ldots ,R_{P+1}\right\}$ , Baseline Force $F_{0}$

2: **Parameters:** Trigger factor λ ; alignment tolerance $\alpha_{\mathrm{tol}}\geq 1/\sqrt{2}$

3: $T_{\mathrm{mmf}}\leftarrow \lambda F_{0}$ , $L_{\mathrm{stable}}\leftarrow 0$

4: **while** not converged **do**
5:    Find highest energy image, $R_{\text{climb}}$ (index k )
6:    Calculate tangents ${\hat{\tau }}_{i}$ and NEB forces $F^{\text{NEB}}$

7:    Calculate climbing image force $F_{CI}=\|F_{\text{climb}}\|$

8:    **if** $k==k_{\mathrm{prev}}$ **then**
9: $L_{\mathrm{stable}}\leftarrow L_{\mathrm{stable}}+1$

10:    **else**
11: $L_{\mathrm{stable}}\leftarrow 0$ ; clear cached eigenvector
12: **end if**
13: **if** $L_{\mathrm{stable}}\geq \kappa$ **and** $F_{CI}<T_{\mathrm{mmf}}$ **then**
14:   Save $R_{k}$ ; initialize Dimer with cached eigenvector (or ${\hat{\tau }}_{k}$ )
15:   Run Dimer: optimize $\hat{d}$ ; abort if $\alpha <\alpha_{\mathrm{tol}}$ or curvature >0

16:   **if** curvature >0 **then** ⊳ Positive curvature: restore
17: $R_{k}\leftarrow$ saved position; clear cache
18:   **else if** $F_{\mathrm{new}}<F_{CI}$ **then**   ⊳ MMF helped Equation 15.
19: $\alpha \leftarrow |\hat{d}\cdot {\hat{\tau }}_{k}|$

20: $T_{\mathrm{mmf}}\leftarrow F_{\mathrm{new}}\cdot \left(0.5+0.4\;F_{\mathrm{new}}/F_{CI}\right)$

21:     Cache $\hat{d}$ for warm-start
22:     Reparameterize P along arc length; reset optimizer
23:  **else**    ⊳ Failure: linear penalty (Equation 13)
24: $\alpha \leftarrow |\hat{d}\cdot {\hat{\tau }}_{k}|$

25: $T_{\mathrm{mmf}}\leftarrow F_{0}\;\lambda \;\left(\frac{1}{2}+\frac{1}{2}\;\alpha \right)$

26:   **end if**
27:  **else**
28:    Take NEB optimization step on P

29:  **end if**
30: $k_{\mathrm{prev}}\leftarrow k$

31: **end while**

**FIGURE 2:**
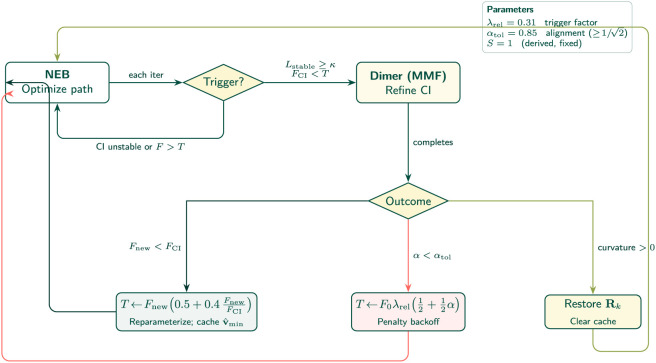
OCI-NEB control flow. After each NEB iteration, the trigger condition gates MMF activation. The dimer outcome determines the next action: on success $\left(F_{\text{new}}<F_{\text{CI}}\right)$ , the threshold adapts downward, and the path is reparameterized; on alignment failure $\left(\alpha <\alpha_{\text{tol}}\right)$ , the linear penalty raises the threshold; on positive curvature, the climbing image is restored to its pre-MMF position. All three branches return the control to the NEB.

Figure 2 summarizes the control flow, showing the three-way outcome branch after each MMF invocation.

##### 2.3.8.1 Configuration

The OCI-NEB implementation exposes tunable parameters that control the aggressiveness of the dimer search. Table 1 lists these parameters alongside their corresponding configuration keys and default values used in this work.

**TABLE 1:** OCI-NEB configuration parameters. Two user-facing parameters control the algorithm; the remaining constants are derived or fixed. The alignment tolerance $\alpha_{\mathrm{tol}}$ has a principled minimum $1/\sqrt{2}$ from the force inversion alignment bound (Figure 1); the reported value 0.85 provides a margin above this minimum. The stability count (κ=5) , maximum MMF steps $\left(N_{\mathrm{mmf}}=1000\right)$ , and penalty shape (S=1) are fixed internal constants.

Parameter description	Symbol	Configuration Key	Value
Relative trigger factor	$\lambda_{\mathrm{rel}}$	ci_mmf_after_rel	0.31
Alignment tolerance	$\alpha_{\mathrm{tol}}$	ci_mmf_angle	0.85

The remaining internal constants— thestability count κ=5 , MMF step budget $N_{\mathrm{mmf}}=1000$ , and penalty shape S=1 (Equation 13)—are fixed. On successful operation, the converged dimer eigenvector is cached for warm-starting the subsequent MMF calls. Both user-facing parameters were held constant across the Baker–Chan and OptBench Pt (111) benchmarks without system-specific tuning; the performance analysis is provided in Section 4.

### 2.4 Computational details

All calculations use the eOn software package (Goswami et al., 2026)^2^. The metatomic interface (Bigi et al., 2025) to the PET-MAD-S v1.5.0 machine learning interatomic potential (Mazitov et al., 2025a; Mazitov et al., 2025b) provides energy and forces. This non-equivariant point-edge transformer model is trained on the diverse MAD dataset, encompassing bulk crystals, surfaces, and molecular fragments at the PBEsol functional level. A Snakemake (Mölder et al., 2021) workflow orchestrated concurrent runs on a single machine with an AMD Ryzen Threadripper PRO 5945WX (24 core and 48 threads) and an NVIDIA T400 GPU with 4 GB VRAM. For efficient concurrency on the singular GPU, we used the NVIDIA multi-processing service. As per the established best practices (Kästner and Sherwood, 2008; Goswami, 2025a) we use the limited-memory Broyden–Fletcher–Goldfarb–Shanno (Liu and Nocedal, 1989) for the translation steps of the dimer and the conjugate gradient optimizer (Chapra and Canale, 2015) for its rotation steps. All reported gradient evaluation counts represent the total potential energy surface queries, inclusive of those incurred during dimer rotation steps, as tracked by the global force call counter in the eOn solver.

We assess the performance of the OCI-NEB algorithm against the standard CI-NEB method across the 25 reactions of the Baker–Chan transition-state test suite (Baker and Chan, 1996). This covers broad chemical archetypes, including systems undergoing dissociations ( ${\text{H}}_{2}\text{CO}$ ), insertions (silene), ring-opening (cyclopropyl), and rotational transitions (acrolein). Since these systems exhibit diverse PES features, ranging from stiff covalent bonds to weak intermolecular forces, they provide a rigorous test for the adaptability and robustness of OCI-NEB compared to the standard CI-NEB protocol.

The Baker–Chan endpoint geometries use the atom ordering convention specified in the original test set (Baker and Chan, 1996), where corresponding atoms share the same index across the reactant and product. This ordering is preserved by the readcon-core implementation^3^ of the v2 con file specification, which maintains fifth-column atom-type assignments on loading. Preserving this ordering eliminates atom-matching heuristics that can introduce spurious permutations in the initial path.

Both methods utilized identical initialization parameters (for the Baker–Chan, S-IDPP (Schmerwitz et al., 2024), eight intermediate images) and convergence criteria (0.05eV/Å) . Baseline CI-NEB and the OCI-NEB protocols utilized identical optimization backends and convergence criteria. The specific hyperparameters governing the hybrid OCI-NEB triggers are detailed in Table 2.

**TABLE 2:** Optimization hyperparameters. Shared parameters apply to both methods. OCI-NEB-specific parameters govern the activation and stability of the dimer. These default values demonstrate robustness across diverse chemical archetypes. Section 4 contains a detailed performance analysis of the most influential parameters, specifically, the trigger thresholds and alignment tolerances.

Category	Parameter	Symbol	Value
Shared	Potential	V(R)	PET-MAD-S v1.5.0
	Optimizer	-	LBFGS
	Convergence force	$F_{\mathrm{tol}}$	0.05 eV/Å
	Images	$N_{\mathrm{img}}$	8
	Spring constant	$k_{sp}$	1–10 eV/Å**2 (energy weighted)
	CI activation (relative)	$\lambda_{CI}$	0.8
	S-IDPP growth factor	$\alpha_{\mathrm{idpp}}$	0.33
OCI-NEB	MMF relative trigger	$\lambda_{\mathrm{rel}}$	0.31
	Alignment tolerance	$\alpha_{\mathrm{tol}}$	0.85
	Dimer rot. convergence	$\phi_{\mathrm{tol}}$	10.0°

Both methods converge for all systems, and the OCI-NEB strictly improves performance in every case, so we use a hierarchical Bayesian negative binomial regression with varying slopes modeled via B-splines with an intercept for each system for quantifying the performance as a function of the distance of the initial path saddle estimate to the final configuration (Goswami and Jónsson, 2025; Goswami, 2025a; Goswami, 2025b). Distances (Goswami, 2025c) used involve permutation corrections through the iterative rotations and assignments (IRA) algorithm (Gunde et al., 2021). The SI contains more details.

Many methods use synthetic benchmarks without considering the broader applicability of the underlying algorithms. The NEB applies to gas-phase molecular systems and extended systems in catalysis alike. We use the OptBench (Chill et al., 2014) Pt (111) heptamer island benchmark, which focuses on metallic surface diffusion. This dataset contains 59 low-energy mechanisms for the rearrangement of a platinum heptamer on a Pt (111) slab. Each system comprises 343 atoms: seven adatoms on a fcc Pt (111) slab model composed of six atomic layers, three out of which are kept frozen to represent the bulk system. To strictly adhere to the benchmark definition, these calculations use an analytic Morse potential (morse_pt) rather than the MLIP. The setup involved five intermediate images initialized via linear interpolation, without energy-weighted springs, and a fixed spring constant of five. We enforced a tighter convergence criterion of 0.001 eV/Å for the norm of the force vector. Frozen atoms in the bottom slab eliminate the rotational and translational degrees of freedom, so explicit removal of these modes is unnecessary. The OCI-NEB and CI-NEB parameters are otherwise identical to those given in Table 2.

## 3 Results

The OptBench Pt (111) heptamer benchmark probes the efficiency in the regime of solid-state surface diffusion. Table 3 summarizes the performance statistics. Despite the use of a simple linear initialization and a tighter convergence criterion (0.001 eV/Å) , OCI-NEB demonstrates improved efficiency. OCI-NEB reduced the mean computational cost by 31%, decreasing from 409 to 280 evaluations. Of the 59 mechanisms, 52 show improvement, while seven exhibit modest regressions (up to 18% increased cost), all on systems where the dimer triggers near a shallow basin. The average RMSD between the final saddle configurations is $6.8\times 10^{-5}\text{Å}$ with negligible energetic differences, confirming that OCI-NEB locates the same transition states as CI-NEB on this benchmark.

**TABLE 3:** Statistical summary of the Pt (111) heptamer island benchmark. Values represent gradient evaluations across the 59 diffusion mechanisms. The same parameters ( $\lambda_{\mathrm{rel}}=0.31$ and $\alpha_{\mathrm{tol}}=0.85$ ) are used without system-specific tuning; seven of the 59 systems show modest regressions.

Metric	CI-NEB	OCI-NEB	Reduction
Mean	409	280	31%
Median	357	311	13%
Min	172	92	47%
Max	1,187	917	23%

While the OptBench Pt (111) heptamer benchmark demonstrates efficiency gains for solid-state surface transitions, the Baker–Chan set focuses on molecular reactions in the gas phase. Table 4 summarizes the computational cost for each system in the Baker-Chan benchmark. OCI-NEB demonstrates a consistent advantage: a 2.44× overall speedup (13,920 vs. 5,712 total force evaluations; median per-system ratio 2.20× ). OCI-NEB strictly improves the performance on all 24 systems with 0/24 regressions, with speedup ratios ranging from 1.43× to 8.76× . The reported parameters were determined via an optuna-based sensitivity study using tree-structured Parzen estimation (Bergstra et al., 2011) (Figure 7).

**TABLE 4:** Comparison of the total force evaluations for CI-NEB and OCI-NEB on the Baker test set. *Diff* indicates the reduction in evaluations achieved by OCI-NEB. *RMSD* is the IRA-corrected root-mean-square displacement between the CI-NEB and OCI-NEB saddle-point geometries.

ID	Reaction	CI-NEB	OCI-NEB	Diff	RMSD (Å)
01	HCN→HNC	698	179	519	$3.6\times 10^{-4}$
02	$\text{HCCH}\to {\text{CCH}}_{2}$	250	163	87	$6.9\times 10^{-4}$
03	${\text{H}}_{2}\text{CO}\to {\text{H}}_{2}+\text{CO}$	562	259	303	$2.2\times 10^{-3}$
04	${\text{CH}}_{3}\text{O}\to {\text{CH}}_{2}\text{OH}$	242	108	134	$3.3\times 10^{-3}$
05	cyclopropyl ring opening	170	114	56	$2.2\times 10^{-3}$
06	bicyclo [1.1.0]butane → *trans*-butadiene	570	345	225	$5.9\times 10^{-3}$
08	formyloxyethyl 1,2-migration	434	164	270	$5.9\times 10^{-2}$
09	parent Diels-Alder cycloaddition	922	238	684	$1.3\times 10^{-2}$
10	s-tetrazine $\to 2\text{HCN}+{\text{N}}_{2}$	442	144	298	$1.1\times 10^{-2}$
11	*trans*-butadiene → *cis*-butadiene	218	144	74	$3.6\times 10^{-3}$
12	${\text{CH}}_{3}{\text{CH}}_{3}\to {\text{CH}}_{2}{\text{CH}}_{2}+{\text{H}}_{2}$	666	258	408	$2.5\times 10^{-3}$
13	${\text{CH}}_{3}{\text{CH}}_{2}\text{F}\to {\text{CH}}_{2}{\text{CH}}_{2}+\text{HF}$	322	144	178	$2.7\times 10^{-3}$
14	acetaldehyde keto-enol tautomerism	274	159	115	$3.4\times 10^{-3}$
15	HCOCl→HCl+CO	434	149	285	$2.8\times 10^{-3}$
16	${\text{H}}_{2}\text{O}+{{\text{PO}}_{3}}^{-}\to {\text{H}}_{2}{{\text{PO}}_{4}}^{-}$	1,034	721	313	$5.5\times 10^{-3}$
17	${\text{CH}}_{2}{\text{CHCH}}_{2}{\text{CH}}_{2}\text{CHO}$ Claisen rearrangement	866	451	415	$1.1\times 10^{-2}$
18	${\text{SiH}}_{2}+{\text{CH}}_{3}{\text{CH}}_{3}\to {\text{SiH}}_{3}{\text{CH}}_{2}{\text{CH}}_{3}$	778	322	456	$5.2\times 10^{-2}$
19	HNCCS→HNC+CS	834	413	421	$6.6\times 10^{-3}$
20	${{\text{HCONH}}_{3}}^{+}\to {{\text{NH}}_{4}}^{+}+\text{CO}$	674	210	464	$8.0\times 10^{-3}$
21	acrolein rotational TS	322	191	131	$2.0\times 10^{-2}$
22	HCONHOH→HCOHNHO	330	161	169	$1.8\times 10^{-2}$
23	$\text{HNC}+{\text{H}}_{2}\to {\text{H}}_{2}\text{CNH}$	1,586	181	1,405	$6.7\times 10^{-3}$
24	${\text{H}}_{2}\text{CNH}\to {\text{HCNH}}_{2}$	394	184	210	$2.6\times 10^{-3}$
25	${\text{HCNH}}_{2}\to \text{HCN}+{\text{H}}_{2}$	898	310	588	$8.5\times 10^{-3}$
​	Mean	580.0	238.0	342.0	$1.2\times 10^{-2}$
​	Median	502.0	182.5	291.5	$5.9\times 10^{-3}$

**FIGURE 3:**
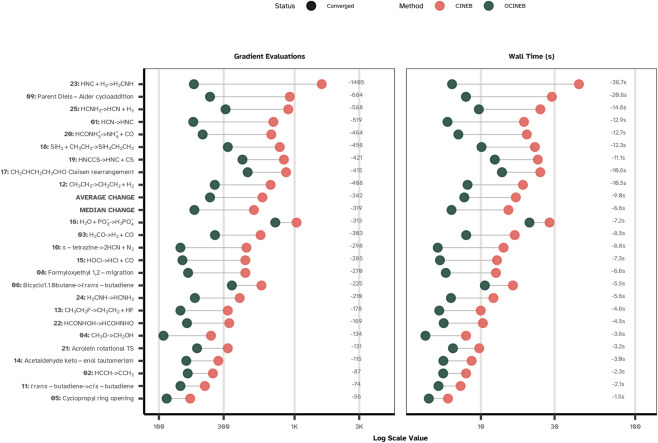
Comparative computational cost for the test set of transition configurations (Baker and Chan, 1996). The “dumbbell” spans illustrate the reduction in the gradient evaluations (left) and wall-clock time (right) achieved by OCI-NEB (teal) relative to CI-NEB (coral).

Figure 3 visualizes the system-specific efficiency gains. The breakdown demonstrates that the performance differential varies across the test set although the OCI-NEB strictly performs better for every system. For straightforward rearrangements (e.g., 05 and 06), both methods perform comparably. For systems exhibiting larger structural reorganizations or flatter potential energy landscapes (e.g., 23, 09, and 01), the gap widens. The “dumbbell” spans in Figure 3 illustrate these reductions; OCI-NEB prevents the cost blowouts in the sections of configuration space with near-zero forces.

**FIGURE 4:**
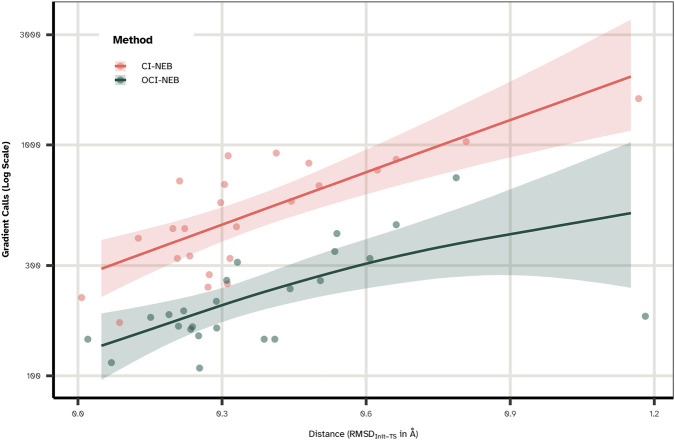
Algorithmic robustness profile modeled via Bayesian negative binomial regression. The plot tracks the predicted computational cost (gradient calls and log scale) as a function of the initial structural displacement from the final transition state. Shaded regions indicate 95% credible intervals. Both methods show a log-linear rise in cost with distance, but OCI-NEB (teal) maintains a consistent advantage of efficiency over CI-NEB (coral), demonstrating that the MMF acceleration effectively lowers the computational overhead across the search space.

Figure 4 illustrates the dependence of computational effort on the quality of the initial guess. Both the algorithms exhibit a log-linear increase in the energy and gradient evaluations as the initial structural displacement grows, which is a consequence of the chain-of-states methods, where distal starting points require more iterations to drag the elastic band toward the saddle. OCI-NEB establishes a consistent efficiency offset, operating strictly below the cost trajectory of CI-NEB.

In the near-harmonic regime around the transition state, the RMSD between the initial estimate and the final configurations is low; therefore, the credible intervals for the methods overlap (Figure 4), which is consistent with parity.

When the initial path already lies close to the converged NEB path, convergence can occur before the MMF trigger fires. As the displacement increases, the advantage of the hybrid protocol becomes clearer. The MMF phase allows the climbing image to deviate slightly from the local tangent and moves without having to move the other images of the band. This lowers the pre-factor of the cost scaling and keeps OCI-NEB at a lower computational burden even as the search space expands.

**FIGURE 5:**
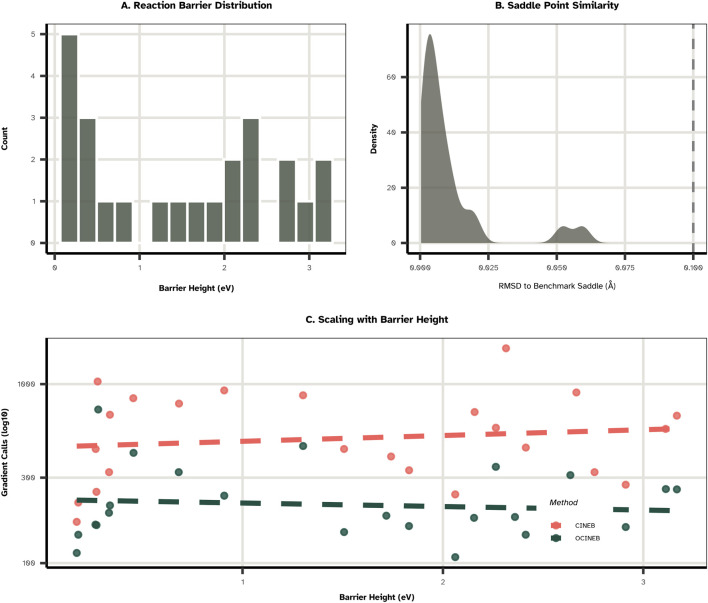
Dataset characterization and drivers of cost. **(A)** Distribution of barrier heights in the test set. **(B)** Density plot of the structural deviation of the OCI-NEB transition states compared to the CI-NEB saddle points; the density peaks below 0.1 Å, confirming correct convergence and equivalent structures. **(C)** Scatter plot of computational cost vs. barrier height. The lack of a strong trend contrasts with the clear scaling seen in Figure 4, indicating that the initial structural guess quality drives the cost more than the energetics of the reaction.

To further contextualize these performance gains, Figure 5 contrasts the structural difficulty with energetic difficulty. Panel C indicates a lack of correlation between the reaction barrier height and the computational cost. While one might expect higher barriers to require more energy and gradient evaluations, the data indicates that the distance of the saddle configuration from the reactant (Figure 4) is the primary driver of the optimization effort. Panel B confirms that this efficiency does not come at the cost of accuracy, and the OCI-NEB identifies transition states with a structural deviation of approximately 0.01 Å from the CI-NEB reference in almost every case. This indicates that the “stiffness” of the optimization problem derives more from the “memory” of the initial path than the height of the hill, reinforcing the value of OCI-NEB decoupling from the neighboring images.

**FIGURE 6:**
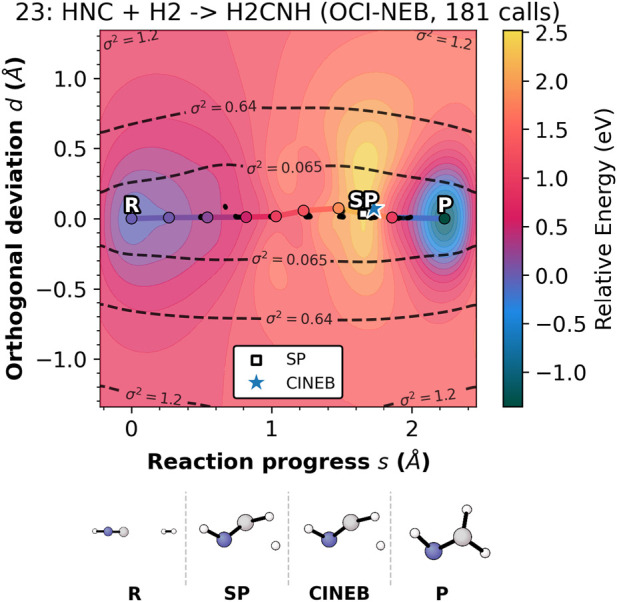
2D reaction landscape for $\text{HNC}+{\text{H}}_{2}\to {\text{H}}_{2}\text{CNH}$ (system 23, 8.76× speedup). The background contour is a derivative Gaussian process interpolation of the OCI-NEB optimizer history using an inverse multiquartic kernel (Goswami, 2025c). Black dots: all images sampled during OCI-NEB. Colored circles: final converged path. White square: OCI-NEB saddle point (SP). Blue star: CI-NEB saddle point. Molecular structures at key configurations are shown below the landscape.

The largest RMSD between the CI-NEB and OCI-NEB saddle points across the 24 systems is 0.059 Å (formyloxyethyl, System 08), with a median of 0.006 Å (Table 4). All deviations are sub-angstrom, confirming that OCI-NEB converges to the same transition states as CI-NEB. Figure 6 illustrates the largest speedup in the benchmark: the $\text{HNC}+{\text{H}}_{2}\to {\text{H}}_{2}\text{CNH}$ reaction (system 23, 8.76× ), where OCI-NEB converges in 181 evaluations compared to 1,586 for CI-NEB. The 2D projections (Goswami, 2025c) for all 24 systems are provided in Supplementary Information.

## 4 Discussion

The relationship between the initial guess of the path and the computational cost demonstrates the advantage for the dimer acceleration. As shown in Figure 4, the scaling profiles diverge as the initial root-mean-square deviation of atomic positions distance to the transition state $\left(RMSD_{I,S}\right)$ increases. For good estimates of the initial saddle configuration ( $RMSD_{I,S}<0.3$ Å), the performance remains parity-bound. In the regime of poor initialization ( $RMSD_{I,S}>0.6$ Å), CI-NEB exhibits a steep efficiency penalty, whereas OCI-NEB maintains a consistently lower cost trajectory. This confirms that the decoupling of the reaction path allows the dimer method to recover efficiently from poor starting geometries. The relation between the distance to the point of interest and improvements from acceleration follows the trends of other methods (Goswami et al., 2025b). In all cases, we expect systems that undergo more iterations to have better gains from such techniques.

The alignment check $\alpha >\alpha_{\mathrm{tol}}$ serves two purposes, namely, it ensures that the dimer’s minimum mode is sufficiently aligned with the NEB tangent to maintain Householder stability, and it prevents the dimer from wandering to a saddle unrelated to the reaction coordinate. Even when α is high, the dimer may translate far from the initial climbing-image position, potentially finding a different first-order saddle. The adaptive penalty (Equation 13) and the force-based success criterion $\left(F_{\mathrm{new}}<F_{CI}\right)$ limit such excursions: if the dimer moves off-path without reducing the force, the threshold is raised, and the NEB resumes control. After a successful dimer step, the arc-length reparameterization redistributes images evenly along the updated path, preventing the band from becoming stretched. This combination ensures the dimer accelerates convergence toward the saddle of interest rather than diverging to unrelated stationary points. Starting the MMF early can lead to a separate and higher energy saddle when seeded with a poor initial path. We consider the keto–enol tautomerism of vinyl alcohol, 14_vinyl_alcohol, where the initial conditions of the indexing in the reactant and product change calculations markedly. The IRA (Gunde et al., 2021) algorithm in this instance, when applied to the endpoints, re-orders the H atom to drive a 1,2-hydrogen shift along the C–C bond. Although energetically unfavorable, this permutation yields a lower Euclidean distance than the chemically correct 1,3-arch. An initial path generated from the permuted endpoints forces the hydrogen atom through the dense electron density of the C–C bond, creating a massive artificial steric barrier. Purely geometric alignment metrics cannot handle such situations, though for many cases including this one, masking the hydrogen atoms and aligning the heavier elements before using the constrained shortest distance assignments (CShDA) or point group symmetry measures (Gunde et al., 2024) can fix the ordering concerns. To maintain our focus on the OCI-NEB, we report results based on the coordinates from the initial structures without alignment, as the endpoint alignment forms part of the initial path considerations not covered here but actively worked on for a follow-up.

The OCI-NEB scheme exposes two user-facing parameters, namely, $\lambda_{\mathrm{rel}}$ , which governs when the NEB-to-dimer handover occurs, and $\alpha_{\mathrm{tol}}$ , whose lower bound $1/\sqrt{2}$ follows from the force inversion alignment condition. The penalty shape S=1 is fixed by the uniqueness of the linear interpolation (Equation 12).

The reported parameters were identified through a systematic sensitivity study using the Optuna hyperparameter optimization framework (Akiba et al., 2019). The optimizer utilizes the Tree-structured Parzen Estimator (TPE) (Bergstra et al., 2011), a sequential model-based algorithm that differs from Gaussian process Bayesian optimization in a fundamental way; rather than modeling the objective as a function of parameters p(y∣x) , TPE separately models the parameter distributions conditioned on performance. Specifically, it partitions observations into “good” (below a quantile threshold $y^{*}$ ) and “bad” groups, fitting separate densities $l\left(x\right)=p\left(x|y<y^{*}\right)$ and $g\left(x\right)=p\left(x|y\geq y^{*}\right)$ . The expected improvement criterion then reduces to a ratio of these densities:

$$EI\left(x\right)\propto \frac{l\left(x\right)}{g\left(x\right)}.$$ (16)

This formulation avoids the cubic scaling of Gaussian process fitting and handles conditional, categorical, and Tree-structured parameter spaces without modification, which suits the mixed parameter types in OCI-NEB.

Each of the 200 trials evaluates a candidate parameter vector by running OCI-NEB on all 24 Baker systems and summing the total force evaluations. No regressions are hidden by a subset selection. A MedianPruner terminates the unpromising trials early when their intermediate results exceed the median of the completed trials at the same step, reducing the total computational budget without biasing the final parameter estimates.

To quantify the contribution of each parameter to the overall performance, we apply the functional ANOVA (fANOVA) decomposition (Hutter et al., 2014). The fANOVA framework decomposes the predicted objective function into additive components:

$$f\left(x\right)=f_{0}+\underset{i}{\sum }f_{i}\left(x_{i}\right)+\underset{i<j}{\sum }f_{ij}\left(x_{i},x_{j}\right)+\cdots \;,$$ (17)

where $f_{0}$ is the grand mean, $f_{i}\left(x_{i}\right)$ captures the marginal effect of parameter i , and $f_{ij}\left(x_{i},x_{j}\right)$ captures pairwise interactions. The fraction of total variance attributable to each term identifies which parameters genuinely drive the performance and which have no effect.

**FIGURE 7:**
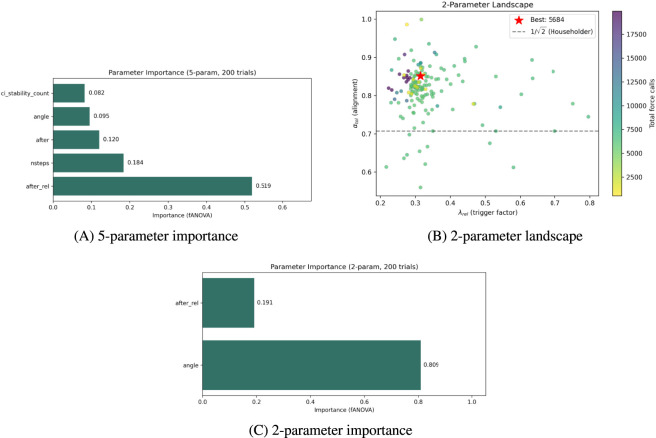
Optuna sensitivity study (200 trials, TPE sampler, all 24 Baker systems). **(A)** Five-parameter fANOVA importance: $\lambda_{\mathrm{rel}}$ dominates, justifying the fixation of three minor parameters as internal constants. **(B)** Two-parameter landscape: total force calls (color) as a function of $\lambda_{\mathrm{rel}}$ and $\alpha_{\mathrm{tol}}$ . The dashed line marks the alignment bound $\alpha_{\mathrm{tol}}=1/\sqrt{2}$ ; trials below this line incur high cost. The red star marks the optimum at (0.31, 0.85). **(C)** Two-parameter fANOVA importance: with noise parameters removed, $\alpha_{\mathrm{tol}}$ accounts for 81% of the variance.

The fANOVA importance analysis proceeds in two stages. In the initial 5-parameter study (Figure 7, panel A), $\lambda_{\mathrm{rel}}$ accounts for 52% of the total variance, while $\alpha_{\mathrm{tol}}$ , the absolute trigger floor, the MMF step budget, and the stability count each contribute less than 20%. This hierarchical structure motivates fixing the three least important parameters as internal constants, thus reducing the user-facing space to two parameters.

A second 200-trial study over the reduced ( $\lambda_{\mathrm{rel}}$ , $\alpha_{\mathrm{tol}}$ ) space reveals the complementary picture (Figure 7, panels B–C). With the noise parameters removed, $\alpha_{\mathrm{tol}}$ now accounts for 81% of the variance: the alignment tolerance is the primary sensitivity driver among the two retained parameters. The contour landscape (panel B) shows the Householder stability bound $\alpha_{\mathrm{tol}}=1/\sqrt{2}$ as a dashed line; trials below this bound incur high cost (light/yellow markers). The optimal basin concentrates near ( $\lambda_{\mathrm{rel}}\approx 0.31$ , $\alpha_{\mathrm{tol}}\approx 0.85$ ), with the best trial at 5,684 total force calls (red star). The margin between the theoretical minimum $1/\sqrt{2}\approx 0.707$ and the empirical optimum 0.85 absorbs the dimer-rotation approximation error far from the saddle.

**FIGURE 8:**
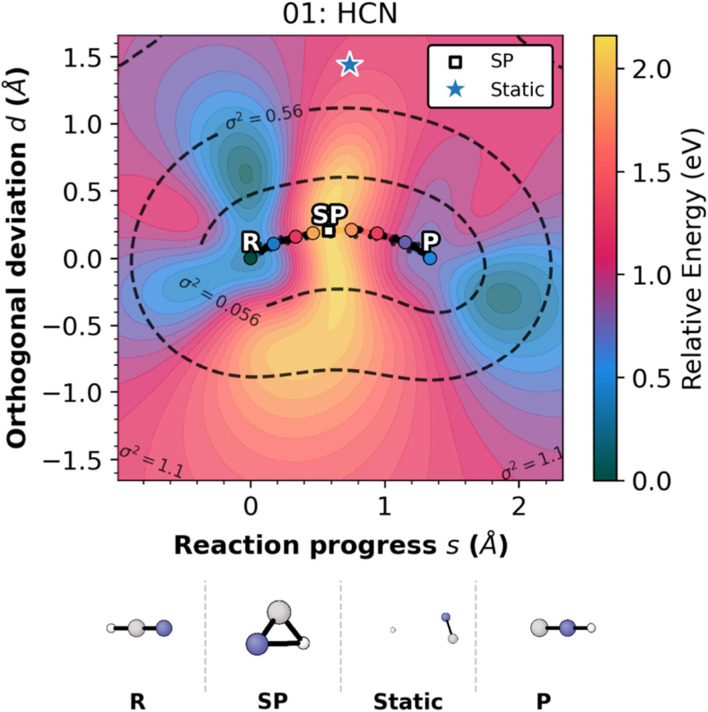
Static handover failure on HCN (System 01). 2D RMSD projection (Goswami, 2025c). SP (white square): CI-NEB saddle point; the OCI-NEB saddle (179 calls) coincides with SP at this scale (RMSD <0.001 Å) and is omitted for clarity. Static (orange star): dimer endpoint from the static handover protocol (531 total calls), which drifts to an unrelated saddle (travel RMSD 0.885 Å) far off the reaction path. The fixed 0.5 eV/Å threshold triggers the dimer prematurely, and the dimer follows the local lowest mode to an irrelevant stationary point. OCI-NEB avoids this failure via the adaptive trigger and alignment check.

The “static handover” approach, corresponding to the protocol of Ásgeirsson et al. (2021) and forming the basis of the automated workflow of Park J. et al. (2025), applies a single fixed force threshold (0.5 eV/Å) for the NEB-to-dimer transition without adaptive back-off. Across all 24 Baker systems, the static protocol requires 8,360 total force evaluations compared to 5,712 for OCI-NEB—a 46% overhead (Supplementary Table S2). On System 01 (HCN, Figure 8), the static protocol converges to the wrong saddle point (dimer travel RMSD 0.885 Å, status BAD), while OCI-NEB finds the correct transition state in 179 calls. On System 16 ( ${\text{H}}_{2}{{\text{PO}}_{4}}^{-}$ ), the static dimer wanders for 1,422 force evaluations (travel RMSD 1.10 Å) before converging. OCI-NEB converges all 24 systems correctly with zero regressions, eliminating the need for manual threshold selection and providing built-in failure recovery via the adaptive penalty. The Claisen system (System 17), where both the methods converge correctly but OCI-NEB is 17% faster (451 vs. 543 calls), is presented in Supplementary Information (Supplementary Figure S8).

The reported parameters ( $\lambda_{\mathrm{rel}}=0.31$ and $\alpha_{\mathrm{tol}}=0.85$ ) emerge from the TPE-based sensitivity study (Figure 7). This configuration converged all 24 Baker systems strictly faster than CI-NEB, with a 2.44× overall speedup and 0/24 regressions. Parallel implementations of the NEB may make stricter protocols more preferable by increasing the stability without wall-time increases; even with parallel implementations, OCI-NEB should yield favorable statistics.

A natural question is why the dimer follows the NEB rather than the reverse. The choice of taking the NEB-CI tangent as the preferred mode over the dimer axis is based on practical considerations. When switching to the NEB after the dimer axis dephases from the NEB-CI tangent, despite reparameterization, if the dimer has moved the image far from the path, many calculations are required to enforce equal spacing, triggering the CI and, eventually, the dimer. A dimer, being an initial point method not tied to other points on the surface, can be started from any point with lower cost. Thus, the dimer follows the NEB and not the other way around, and OCI-NEB provides greater dividends with more images in the path.

The dimer axis may differ from the NEB-CI tangent, even though they both approximate the Hessian’s lowest mode, due to the approximations made in each method. In the CI-NEB, the tangent estimate derives from a weighted average of the vectors to neighboring images from the CI, which has variable spacing based on the springs. The dimer axis derives more directly from the local mode of the Hessian and always has a small finite fixed distance between its images. The dimer axis is more flexible than the NEB-CI tangent, which is damped by the presence of the rest of the band. These separate considerations effectively improve the response of the NEB-CI in terms of the number of calculations needed to track the true minimum mode of the Hessian.

## 5 Conclusion

We present the OCI-NEB algorithm, an adaptive hybrid optimization strategy that integrates the stability of the NEB method with the efficiency of a MMF saddle-point search. The algorithm exposes two parameters: the relative trigger factor $\lambda_{\mathrm{rel}}$ and the alignment tolerance $\alpha_{\mathrm{tol}}$ , whose lower bound $1/\sqrt{2}$ follows from the force inversion alignment condition. The penalty shape ( S=1 and B=0.5 ) is the unique linear member of the admissible family and requires no tuning. After each successful dimer step, an arc-length reparameterization redistributes images evenly along the path at zero force-call cost, thus maintaining the path quality throughout the hybrid optimization. The underlying force inversion structure, shared by both the climbing image and dimer dynamics, ensures that switching between the two phases preserves the shared fixed point at the saddle.

The method assumes an initial path (here, from S-IDPP) with energy-weighted springs providing higher image density near the saddle. By decoupling the climbing image from the band during MMF bursts, OCI-NEB reduces the total number of force evaluations and relaxes the requirement on the number of images bracketing the saddle.

Benchmarking against the standard Baker–Chan transition state set using a modern machine-learned potential, PET-MAD-S v1.5.0 demonstrates the practical utility of this approach. Empirically, OCI-NEB achieves a 2.44× overall speedup in force evaluations compared to CI-NEB, with strict improvement on all 24 systems. More importantly, the method exhibits superior performance even where the standard nudged elastic band and the dimer individually struggle, showing an increase in the performance relative to running the NEB to a fixed tolerance and then switching to the dimer, as previously posited in the literature.

A Bayesian negative binomial regression accounting for the distance from the initial saddle estimate to the final configuration (with permutation-corrected distances) quantifies the expected improvement: OCI-NEB requires 0.43× [95% CrI: 0.36, 0.50] as many gradient evaluations as CI-NEB, corresponding to a 57.4% reduction ( −63.9% , −49.6% ). The model and diagnostics are detailed in Supplementary Information.

The method proves equally effective for surface systems. Tests on the OptBench Pt (111) heptamer island dataset, comprising 59 distinct diffusion mechanisms, confirm that OCI-NEB maintains its efficiency advantage. Despite the tight convergence criteria and the presence of low-frequency modes typical of surface diffusion, the hybrid protocol yielded a 31% mean reduction in force calculations with negligible structural deviation from the CI-NEB saddle points.

These results indicate that OCI-NEB is well-suited to high-throughput transition-state searches, including those driven by machine-learned potentials whose surface topology may deviate from the smooth harmonic basins assumed by traditional optimizers. By dynamically decoupling the saddle point search from the reaction path constraints, OCI-NEB provides a reliable pathway for automated chemical discovery in complex systems. As machine-learned potentials reduce the cost of individual force evaluations, the computational bottleneck shifts toward optimization efficiency on rough landscapes. The method also degrades gracefully to match the CI-NEB results by tuning the relative trigger factor, which controls the degree of path relaxation before dimer activation. These scale-independent parameters transfer across diverse chemical systems without re-tuning, while the penalty shape is derived from the first principles. OCI-NEB, therefore, holds promise for high-throughput calculations, where a single protocol can be applied across a diverse dataset for reaction pathway discovery.

## Data Availability

The datasets presented in this study can be found in online repositories. The names of the repository/repositories and accession number(s) can be found below (on materials cloud archive (Talirz et al., 2020)): https://doi.org/10.24435/materialscloud:ym-my.

## Footnotes

1
https://eondocs.org

2
https://eondocs.org

3
https://lode-org.github.io/readcon-core/spec.html
