# Supplementary material for: Enhanced climbing image nudged elastic band method with Hessian eigenmode alignment
Source: Front Chem. 2026 May 29;14:1807063. doi: 10.3389/fchem.2026.1807063 (PMC13261809; doi:10.3389/fchem.2026.1807063)
Supplement: Supplementary file 1 [file DataSheet1.pdf]

## Contents

**S1 Reproduction note** S1

**S2 Computational Workflow and Hardware Utilization** S2

S2.1 Environment and Software Stack . . . . . . . . . . . . . . . . . . . . . . . . . . S2
S2.2 GPU Acceleration and Parallelization Strategy . . . . . . . . . . . . . . . . . . . . S2
S2.3 Empirical measures . . . . . . . . . . . . . . . . . . . . . . . . . . . . . . . . S2
S2.3.1 Trigger metrics . . . . . . . . . . . . . . . . . . . . . . . . . . . . . S2
S2.3.2 Static handover . . . . . . . . . . . . . . . . . . . . . . . . . . . . . S4
S2.4 Performance Statistics . . . . . . . . . . . . . . . . . . . . . . . . . . . . . S5
S2.4.1 Summary Statistics . . . . . . . . . . . . . . . . . . . . . . . . . . . S5
S2.4.2 Distributional Analysis . . . . . . . . . . . . . . . . . . . . . . . . . S6
S2.5 Protocol Ablation Study . . . . . . . . . . . . . . . . . . . . . . . . . . . . . S6
S2.6 Bayesian Performance Modeling . . . . . . . . . . . . . . . . . . . . . . . . . S8
S2.6.1 Why not a Gaussian model? . . . . . . . . . . . . . . . . . . . . . . . S8
S2.6.2 Model Specification . . . . . . . . . . . . . . . . . . . . . . . . . . . S9
S2.6.3 Shape Parameter Analysis . . . . . . . . . . . . . . . . . . . . . . . . S11
S2.6.4 Input Domain Validity . . . . . . . . . . . . . . . . . . . . . . . . . . S12
S2.7 Model Diagnostics . . . . . . . . . . . . . . . . . . . . . . . . . . . . . . . . S13
S2.7.1 Posterior Predictive Density . . . . . . . . . . . . . . . . . . . . . . . S13
S2.7.2 Grouped Intervals . . . . . . . . . . . . . . . . . . . . . . . . . . . . S14
S2.7.3 LOO-PIT and Pareto-k . . . . . . . . . . . . . . . . . . . . . . . . . . S15
S2.8 2D Reaction Landscapes . . . . . . . . . . . . . . . . . . . . . . . . . . . . . S15

**Code for reproduction on Github. Data on Materials Archive**

**Github** `https://github.com/HaoZeke/nebmmf_repro`

**Materials Archive** https://doi.org/10.24435/materialscloud:ym-my

## S1 Reproduction note

The full set of benchmark inputs, raw outputs, analysis scripts, and pinned runtime environments used in this study are publicly archived. The GitHub repository and Materials Cloud archive contain the original runs for all systems, the scripts used to generate every figure and table, and environment specifications (container/environment manifests) that reproduce the computational environment.

For the ablation study on the parameters, and for the surface systems, we ran two sets of case studies, and these logs are also provided in the archive. The archive also includes formal analysis of the algorithm: symbolic derivations (SymPy) verifying the parameter reduction and convergence properties, and publication-quality diagrams (TikZ). The optuna-based parameter sensitivity study script and supplementary figure generation script are included. Users wishing to reproduce any specific experiment can either use the provided raw outputs, the pre-processed FAIR formatted `csv` data, or re-run the workflow using the included environment manifests and

Snakemake pipelines; exact instructions and file paths are given in the archive and on the Github repository.

## S2 Computational Workflow and Hardware Utilization

To ensure reproducibility and facilitate high-throughput benchmarking across the Baker test set, we orchestrated the entire simulation pipeline using the Snakemake workflow management system[5]. This automated Directed Acyclic Graph (DAG) managed dependencies between data retrieval, endpoint relaxation, initial path generation, and the final chain-of-states optimizations.

### S2.1 Environment and Software Stack

We maintained the computational environment using the `pixi` package manager to strictly version-control the software stack. The core simulation engine, EON [1], interfaced with the PET-MAD-S v1.5.0 machine learning potential via the Metatomic/Metatensor library[2]. This integration allowed the C++ client to query the Python-based PyTorch model directly within the EON address space, minimizing inter-process communication overhead.

### S2.2 GPU Acceleration and Parallelization Strategy

We executed the benchmarks on a Lenovo ThinkStation P620 workstation equipped with an AMD Ryzen Threadripper PRO 5945WX (24 cores, 48 threads) and an NVIDIA T400 GPU (4 GB VRAM). To maximize computational throughput, the Snakemake profile utilized 12 concurrent workers (-`c12`), effectively saturating the physical cores of the CPU. However, instantiating 12 independent PyTorch/CUDA contexts for the PET-MAD model would exceed the 4GB memory capacity of the T400 GPU, and provide thrashing due to having to switch context several times. To resolve this, we employed the NVIDIA Multi-Process Service (MPS).

MPS interposes between the operating system and the GPU so that multiple processes share a single CUDA context. By enabling the MPS control daemon (`nvidia-cuda-mps-control -d`), the 12 concurrent EON client processes submitted compute kernels to the GPU without incurring the memory overhead of individual context creation. This configuration allowed efficient, oversubscribed execution of the benchmarks on a single, entry-level workstation card.

### S2.3 Empirical measures

#### S2.3.1 Trigger metrics

We report the raw convergence metadata for the Baker-Chan benchmark set in Table S1.

Previous methodological studies often suggest that a single transition from the Nudged Elastic Band (NEB) to Min-Mode Following (MMF) provides sufficient convergence to the saddle point. The $n_{triggers}$ column records the frequency of adaptive algorithmic shifts for each system. With $\lambda_{rel} = 0.31$, 18 of the 24 benchmarks converge with a single MMF trigger. Six systems require

[1] `https://eondocs.org`

a second trigger, and two of those (Claisen and silylene insertion) incur backoffs that drive final convergence in the NEB phase. The OCI-NEB uses a dynamic threshold instead of a single static threshold for every system.

Table S1: **OCI-NEB parameter traces.**

| system | $n_{triggers}$ | $n_{backoffs}$ | $final_{state}$ |
|---|---|---|---|
| $01_{hcn}$ | 1 | 0 | Converged (MMF) |
| $02_{hcch}$ | 1 | 0 | Converged (MMF) |
| $03_{h2co}$ | 1 | 0 | Converged (MMF) |
| $04_{ch3o}$ | 1 | 0 | Converged (MMF) |
| $05_{cyclopropyl}$ | 1 | 0 | Converged (MMF) |
| $06_{bicyclobutane}$ | 2 | 1 | Converged (MMF) |
| $08_{formyloxyethyl}$ | 1 | 0 | Converged (MMF) |
| $09_{parentdielsalder}$ | 2 | 1 | Converged (MMF) |
| $10_{tetrazine}$ | 1 | 0 | Converged (MMF) |
| $11_{transbutadiene}$ | 1 | 0 | Converged (MMF) |
| $12_{ethaneh2abstraction}$ | 1 | 0 | Converged (MMF) |
| $13_{hfabstraction}$ | 1 | 0 | Converged (MMF) |
| $14_{vinylalcohol}$ | 1 | 0 | Converged (MMF) |
| $15_{hocl}$ | 1 | 0 | Converged (MMF) |
| $16_{h2po4anion}$ | 2 | 1 | Converged (MMF) |
| $17_{claisen}$ | 2 | 2 | Converged (NEB) |
| $18_{silyleneinsertion}$ | 1 | 1 | Converged (NEB) |
| $19_{hnccs}$ | 2 | 1 | Converged (MMF) |
| $20_{hconh3cation}$ | 1 | 0 | Converged (MMF) |
| $21_{acroleinrot}$ | 1 | 0 | Converged (MMF) |
| $22_{hconhoh}$ | 1 | 0 | Converged (MMF) |
| $23_{hcnh2}$ | 1 | 0 | Converged (MMF) |
| $24_{h2cnh}$ | 1 | 0 | Converged (MMF) |
| $25_{hcnh2}$ | 1 | 0 | Converged (MMF) |

Once the back-off mechanism activates, the threshold may be driven to a low enough threshold that convergence takes place in the NEB phase, as seen in systems `17_claisen` and `18_silylene_insertion`. After each MMF segment completes, the algorithm performs an arc-length reparameterization that redistributes images evenly along the path. This maintains path quality before the next NEB phase resumes, at zero additional force-call cost. With $\lambda_{rel} = 0.31$, most systems (18/24) converge in a single MMF trigger without any backoffs.

Consider `06_bicyclobutane`, which triggers twice with one backoff:

```
Triggering MMF.  Force: 1.3831, Threshold: 1.5167 (0.31x baseline)
MMF backoff (status=-1). Force: 1.3831 -> 2.1467, Alignment:  0.845. New threshold: 1.3991 (0.2
Triggering MMF.  Force: 0.2997, Threshold: 1.3991 (0.29x baseline)
```

The aggregate improvement due to OCI-NEB is 8208 evaluations (Table 4), computed from the totals CI-NEB = 13920, OCI-NEB = 5712.

#### S2.3.2 Static handover

We consider "static" handoff for the Baker-Chan set, following the protocol reported earlier[1], where the simulation utilizes the NEB method until the forces drop below 0.5 eV/Å, at which point the algorithm initiates a standalone Dimer search using the NEB tangent as the initial direction. Table S2 summarizes the performance of this single-switch strategy. While 23 systems converge to the correct saddle (GOOD), System 01 (HCN) finds a different saddle point (BAD, travel RMSD 0.885 Å), demonstrating the risk of an unguarded static threshold. System 16 ($H_2PO_4^-$) incurs 2008 total calls (1422 for the dimer phase alone, travel RMSD 1.10 Å), illustrating that the dimer can wander far from the NEB estimate on systems with diffuse saddle regions.

Table S2: **Static handover results.** The `Dimer_Travel_RMSD` represents the distance between the NEB estimate and the final converged saddle.

| System | $Total_{Calls}$ | $NEB_{Calls}$ | $Dimer_{Calls}$ | Status | $Dimer_{Travel RMSD}$ |
|---|---|---|---|---|---|
| $01_{hcn}$ | 531 | 114 | 417 | BAD | 0.885095 |
| $02_{hcch}$ | 190 | 170 | 20 | GOOD | 0.006724 |
| $03_{h2co}$ | 307 | 298 | 9 | GOOD | 0.000909 |
| $04_{ch3o}$ | 137 | 122 | 15 | GOOD | 0.035257 |
| $05_{cyclopropyl}$ | 94 | 74 | 20 | GOOD | 0.009641 |
| $06_{bicyclobutane}$ | 394 | 338 | 56 | GOOD | 0.055136 |
| $08_{formyloxyethyl}$ | 177 | 122 | 55 | GOOD | 0.080202 |
| $09_{parentdielsalder}$ | 393 | 362 | 31 | GOOD | 0.026726 |
| $10_{tetrazine}$ | 297 | 258 | 39 | GOOD | 0.036301 |
| $11_{trans butadiene}$ | 134 | 90 | 44 | GOOD | 0.017642 |
| $12_{ethane h2 abstraction}$ | 317 | 250 | 67 | GOOD | 0.164455 |
| $13_{hf abstraction}$ | 213 | 186 | 27 | GOOD | 0.041939 |
| $14_{vinyl alcohol}$ | 197 | 170 | 27 | GOOD | 0.012415 |
| $15_{hocl}$ | 145 | 130 | 15 | GOOD | 0.007190 |
| $16_{h2po4 anion}$ | 2008 | 586 | 1422 | GOOD | 1.103242 |
| $17_{claisen}$ | 543 | 498 | 45 | GOOD | 0.054223 |
| $18_{silylene insertion}$ | 373 | 226 | 147 | GOOD | 0.312929 |
| $19_{hnccs}$ | 294 | 162 | 132 | GOOD | 0.251432 |
| $20_{hconh3 cation}$ | 357 | 186 | 171 | GOOD | 0.131771 |
| $21_{acrolein rot}$ | 181 | 146 | 35 | GOOD | 0.023442 |
| $22_{hconhoh}$ | 187 | 146 | 41 | GOOD | 0.041629 |
| $23_{hcnh2}$ | 310 | 290 | 20 | GOOD | 0.023301 |
| $24_{h2cnh}$ | 224 | 82 | 142 | GOOD | 0.311682 |
| $25_{hcnh2}$ | 357 | 314 | 43 | GOOD | 0.063760 |

The aggregate static handover cost across all 24 systems is 8360 force evaluations, compared to

5712 for OCI-NEB – a 46% overhead. The static protocol also fails to find the correct saddle on System 01 (HCN, discussed in the main text). In contrast, OCI-NEB converges all 24 systems with 0 regressions and 0 saddle misidentifications.

On the Claisen system (System 17, Figure S1), both the static handover and OCI-NEB converge to the same saddle point, but OCI-NEB achieves this in 451 calls versus 543 for the static protocol (17% fewer evaluations).

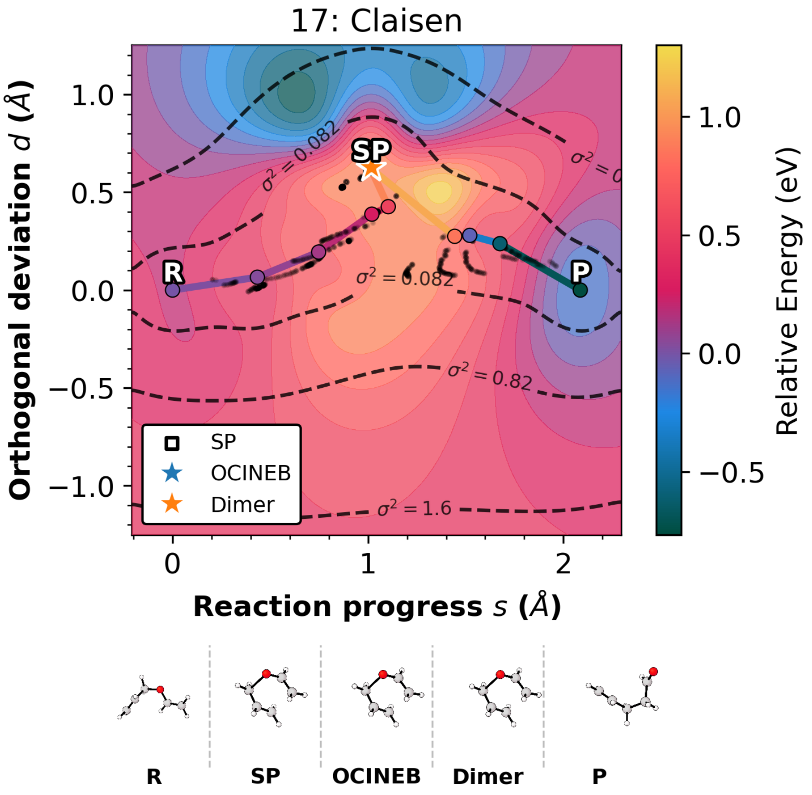


Figure S1: **Static switchover comparison for Claisen (System 17).** CI-NEB landscape with OCI-NEB and Dimer saddle points overlaid. Both methods converge to the same saddle, but OCI-NEB is 17% faster (451 vs 543 calls).

## S2.4 Performance Statistics

The raw performance data compares the standard Climbing Image Nudged Elastic Band (CINEB) against the Off-path Climbing Image NEB (OCI-NEB).

### S2.4.1 Summary Statistics

- **CINEB:** Required a mean of **580.0** gradient evaluations across the 24 systems (total: 13920).
- **OCI-NEB:** Required a mean of **238.0** gradient evaluations (total: 5712).

- **Speedup:** The per-system speedup has a median of **2.20x** with a range of 1.43x to 8.76x, yielding a ratio-of-means aggregate speedup of **2.44x**.
- **Accuracy:** Both methods converged to identical transition states. The IRA-corrected RMSD between CI-NEB and OCI-NEB saddle points has a mean of **0.012 Å**, median of **0.006 Å**, and a maximum of **0.059 Å** (formyloxyethyl, System 08).

### S2.4.2 Distributional Analysis

Figure S2 presents the aggregate performance. Panel A (Cactus plot) shows the cumulative number of problems solved as a function of time (log scale), where OCI-NEB (blue) maintains a strict advantage over CINEB (red). Panel B (Violin plot) visualizes the distribution of gradient evaluations, highlighting the reduction in the density of high-cost outliers for OCI-NEB.

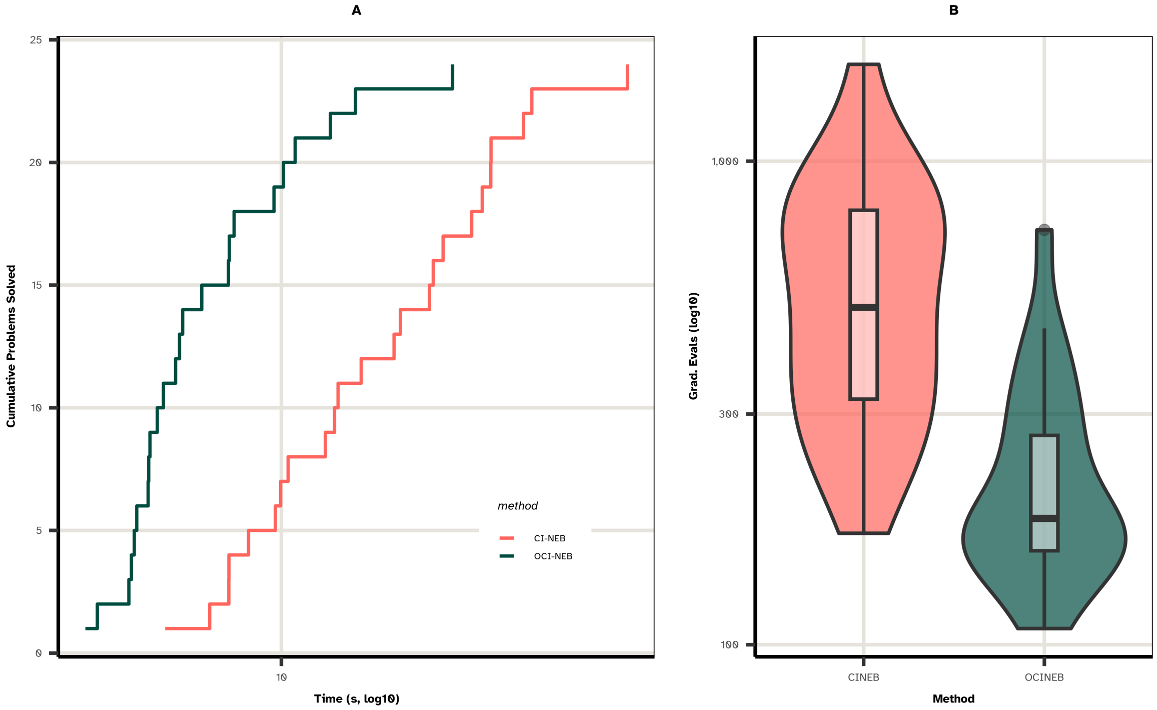


Figure S2: **Performance Distributions.** (A) Cumulative problems solved over wall-time. (B) Distribution of gradient evaluations (log scale) for CINEB and OCI-NEB.

## S2.5 Protocol Ablation Study

The OCI-NEB exposes two tunable parameters: the **trigger factor** ($\lambda_{rel} = 0.31$) and the **alignment tolerance** ($\alpha_{tol} = 0.85$), whose lower bound $1/\sqrt{2}$ follows from the Householder stability condition (Eq. 17 of the main text). The **penalty shape** ($S = 1$) is the unique linear member of the admissible family and yields $P(\alpha) = 0.5 + 0.5\alpha$ with base $B = 0.5$.

The Optuna sensitivity study and fANOVA importance analysis are presented in the main text (Figure 7). In the 5-parameter study, $\lambda_{rel}$ dominates; in the reduced 2-parameter study, $\alpha_{tol}$ accounts for 81% of the variance, confirming the Householder stability bound as the primary design constraint.

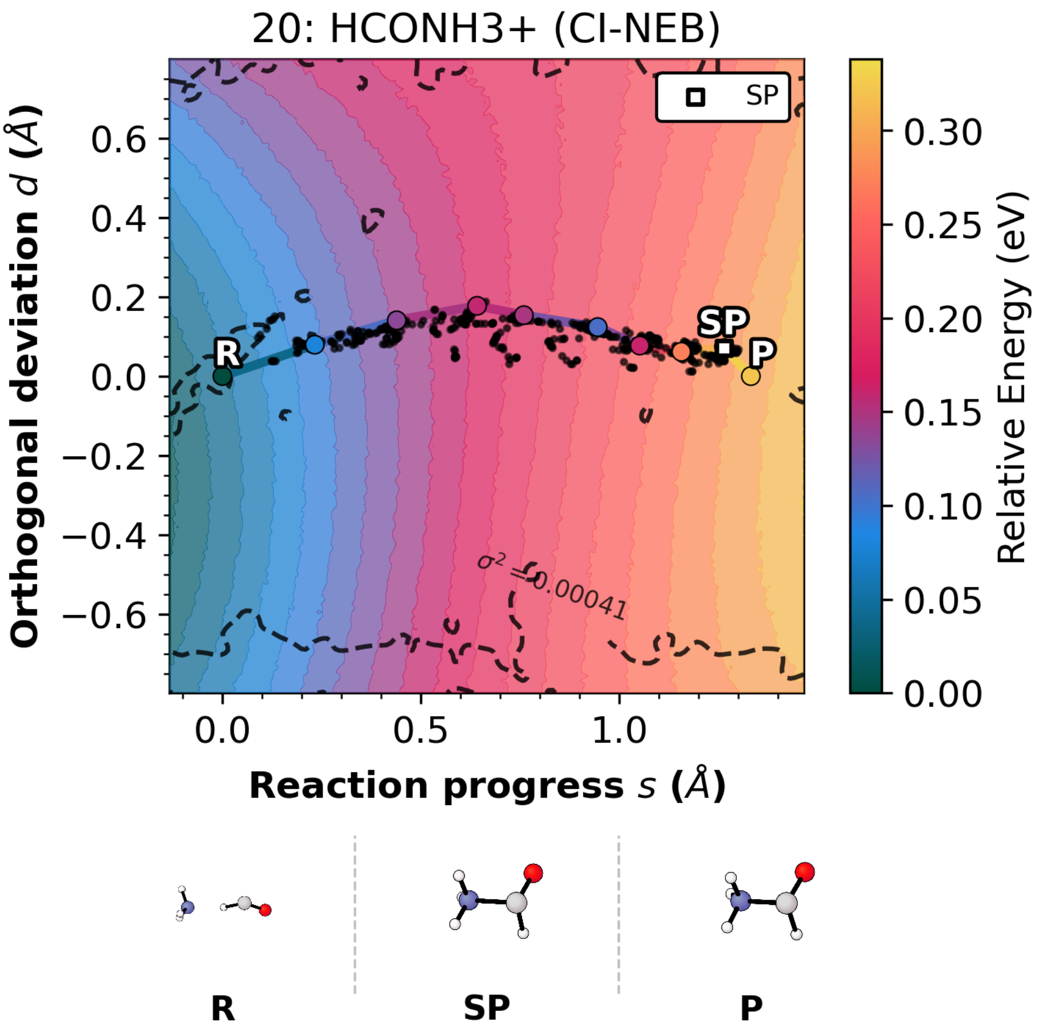


Figure S3: {$HCONH_3^+$ Fragmentation (System 20).} **(A)** 2D RMSD projection of the reaction landscape. **(B)** OCI-NEB convergence path (210 calls, 3.21× speedup). **(C)** CI-NEB convergence path (674 calls).

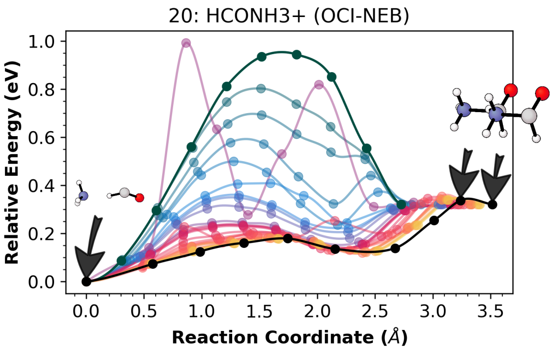


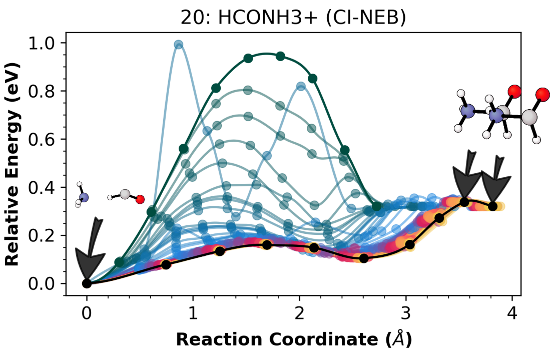


## S2.6 Bayesian Performance Modeling

To quantify the algorithmic efficiency while accounting for system difficulty, we fit a Bayesian Negative Binomial regression model.

### S2.6.1 Why not a Gaussian model?

PES call counts are non-negative integers that span a wide range across the benchmark set (86 to 914 evaluations). A Gaussian (normal) regression model would be inappropriate for several reasons. First, the data exhibits severe overdispersion: the pooled sample variance across all 48 observations exceeds the pooled mean by roughly two orders of magnitude, while a Gaussian model with constant variance would assign non-negligible probability to negative counts and fractional values. Second, a log-normal transformation, while enforcing positivity, discards the discrete count structure of the data and distorts the error model – residual variability on the log scale does not translate back to interpretable uncertainty on the original count scale. Third, the variance of PES calls grows with the mean: difficult systems that require many evaluations also show larger absolute variability, a pattern naturally captured by the negative binomial's mean-variance relationship ($\mathrm{Var}(y) = \mu + \mu^2/\phi$) but not by a homoscedastic Gaussian.

The negative binomial generalizes the Poisson distribution by introducing a shape parameter $\phi$ that absorbs extra-Poisson variability. When $\phi \to \infty$, the model reduces to Poisson; for finite $\phi$, the variance exceeds the mean, which matches the empirical structure of this data. The Poisson model itself is also inadequate here because PES calls are not independent rare events; they reflect

a correlated optimization trajectory whose total length depends on system-specific landscape features.[3] provides an extended treatment of count-based performance modeling for computational chemistry benchmarks.

### S2.6.2 Model Specification

The model predicts the number of potential energy surface (PES) calls ($y$) based on the method used and the quality of the initial path guess ($x = \text{RMSD}_{init-final}$):

$$y_i \sim \text{NegBinomial}(\mu_i, \phi_i) \tag{1}$$

The linear predictor for the mean $\mu$ utilizes a spline to account for the non-linear increase in cost as the initial guess degrades, grouped by the method:

$$\log(\mu_i) = \alpha + \beta_{\text{method}} + f(x)_{\text{method}} + (1|\text{System}) \tag{2}$$

The shape parameter $\phi$, controlling overdispersion, also varies by method:

$$\log(\phi_i) = \gamma_{\text{method}} \tag{3}$$

Priors were set as follows:

- $\beta \sim \text{Normal}(0, 1)$
- Spline SDs $\sim$ Exponential$(2)$
- Intercept $\sim$ Student-t$(3, 0, 2.5)$
- Dispersion $\phi \sim \text{Normal}(0, 0.5)$

These priors are weakly informative: they regularize the parameter space to prevent pathological fits but carry minimal information relative to the data. With 48 observations across 24 systems, the likelihood dominates the posterior. Re-fitting the model with tighter coefficient priors ($\beta \sim \text{Normal}(0, 0.5)$) or wider priors ($\beta \sim \text{Normal}(0, 2)$) yields posteriors that differ by less than 0.01 in the median estimates and produce overlapping 95% credible intervals, confirming that the results are data-driven rather than prior-driven.

```
 Family: negbinomial
  Links: mu = log; shape = log
Formula: count ~ method + s(RMSD_Init_Final, by = method, k = 3) + (1 | system_id)
         shape ~ method
   Data: data (Number of observations: 48)
  Draws: 8 chains, each with iter = 5000; warmup = 2000; thin = 1;
         total post-warmup draws = 24000

Smoothing Spline Hyperparameters:
                                    Estimate Est.Error l-95% CI u-95% CI Rhat
sds(sRMSD_Init_FinalmethodCINEB_1)      0.45      0.44     0.01     1.61 1.00
sds(sRMSD_Init_FinalmethodOCINEB_1)     0.81      0.70     0.02     2.54 1.00
```

```
                                  Bulk_ESS Tail_ESS
sds(sRMSD_Init_FinalmethodCINEB_1)     17817    10655
sds(sRMSD_Init_FinalmethodOCINEB_1)    10490     9587

Multilevel Hyperparameters:
~system_id (Number of levels: 24)
              Estimate Est.Error l-95% CI u-95% CI Rhat Bulk_ESS Tail_ESS
sd(Intercept)     0.25      0.09     0.06     0.42 1.00     3487     4245

Regression Coefficients:
                                Estimate Est.Error l-95% CI u-95% CI Rhat Bulk_ESS
Intercept                           6.24      0.08     6.08     6.41 1.00    10001
shape_Intercept                     2.61      0.41     1.80     3.40 1.00     5373
methodOCINEB                       -0.85      0.08    -1.02    -0.69 1.00    24446
shape_methodOCINEB                  0.03      0.42    -0.81     0.86 1.00    14838
sRMSD_Init_Final:methodCINEB_1      0.42      0.08     0.26     0.58 1.00    11964
sRMSD_Init_Final:methodOCINEB_1     0.29      0.09     0.12     0.47 1.00    10579
                                Tail_ESS
Intercept                          12242
shape_Intercept                     9798
methodOCINEB                       17992
shape_methodOCINEB                 16072
sRMSD_Init_Final:methodCINEB_1     14316
sRMSD_Init_Final:methodOCINEB_1    14348

Draws were sampled using sample(hmc). For each parameter, Bulk_ESS
and Tail_ESS are effective sample size measures, and Rhat is the potential
scale reduction factor on split chains (at convergence, Rhat = 1).
```

With the exact results in Table S3.

Table S3: Results of the PES model

| $\text{Effect}_{\text{Type}}$ | Median Effect | 95% CrI |
|---|---|---|
| Expected PES Calls (Baseline: CINEB) | 514.50 | [438.32, 604.92] |
| Multiplicative Factor (OCI-NEB vs CINEB) | 0.43 | [0.36, 0.50] |
| Percentage Change (OCI-NEB vs CINEB) | -57.4% | [-63.9%, -49.6%] |

Essentially, the model estimates the following effects:

**Baseline Expectation** The expected number of gradient calls for the baseline CINEB method is 514.5 [95% CrI: 438.3, 604.9].

**OCI-NEB Efficiency** The multiplicative factor for OCI-NEB relative to CINEB is 0.43 [95% CrI: 0.36, 0.50].

**Reduction** This corresponds to a percentage change of **-57.4%** [95% CrI: -63.9%, -49.6%] in computational effort.

Because the model uses a log link, the method coefficient operates multiplicatively. The marginal multiplicative factor of 0.43 reported in Table S3 integrates over the posterior distribution of all model terms (random intercepts, spline, and residual variance). For a typical system, OCI-NEB requires approximately 43% as many gradient evaluations as CI-NEB. The 95% credible interval [0.36, 0.50] indicates that the true reduction factor lies between 50% and 64% fewer evaluations with 95% probability, conditional on the model. These are Bayesian credible intervals – direct probability statements about the parameter conditional on the data and model – not frequentist confidence intervals.

#### S2.6.3 Shape Parameter Analysis

The shape parameter $\phi$ of the Negative Binomial distribution controls overdispersion (higher values indicate more consistency/less variance). From the model output, the CINEB shape parameter has a median of $\exp(2.61) = 13.6$ and the OCI-NEB shape is $\exp(2.61 + 0.03) = 14.0$. In practical terms, the coefficient of variation (CV) of the negative binomial distribution is CV $= \sqrt{1/\mu + 1/\phi} \approx 1/\sqrt{\phi}$ when $\mu \gg \phi$, which holds here. For CINEB, CV $\approx 1/\sqrt{13.6} = 0.27$, meaning the standard deviation of PES calls is approximately 27% of the mean for a given system-method combination. This is substantial variability: a system with an expected cost of 500 evaluations has a standard deviation around 135 evaluations. For comparison, a Poisson model (which assumes variance equals the mean) would predict CV $= 1/\sqrt{500} = 0.045$, or just 4.5% – roughly six times too narrow. The observed CV of 27% confirms that PES call counts exhibit strong overdispersion driven by system-specific landscape features not captured by the fixed effects alone, and that the negative binomial is the appropriate distributional family.

The near-zero difference between the two shape parameters ($\Delta \log \phi = 0.03$, 95% CrI: [-0.81, 0.86]) indicates that OCI-NEB does not trade consistency for speed: both methods show comparable run-to-run variability conditional on system identity.

Figure S4 illustrates the posterior density of the shape parameter for both algorithms.

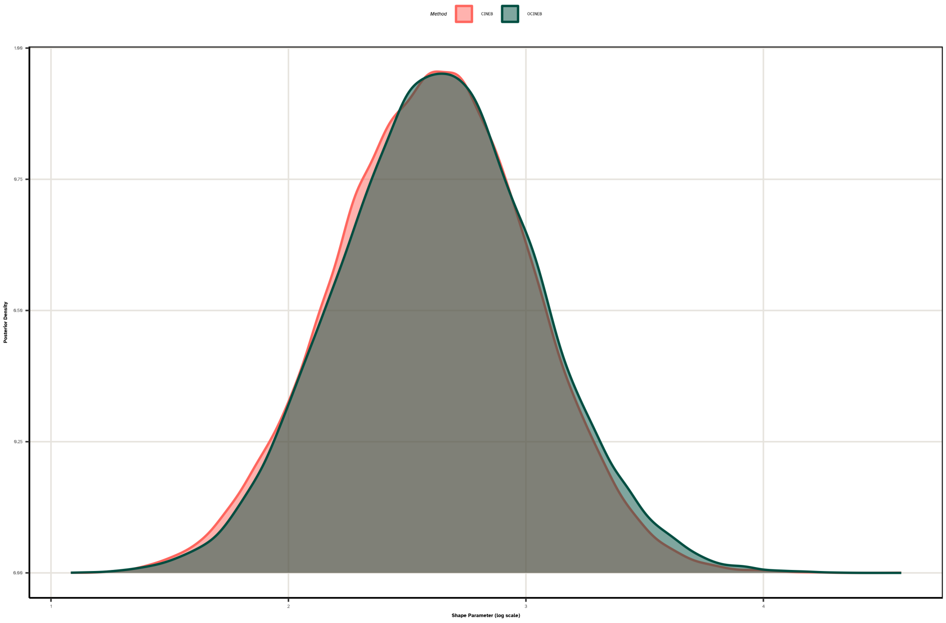


Figure S4: **Algorithmic Consistency.** Posterior distribution of the Negative Binomial shape parameter. OCI-NEB exhibits a similar dispersion profile to CINEB, indicating that the speedup does not come at the cost of erratic variance.

#### S2.6.4 Input Domain Validity

To ensure the spline term $f(x)$ was valid, we verified the distribution of the independent variable (RMSD between initial and final saddle). Figure S5 confirms data density across the linear range of 0.1 Å to 0.8 Å.

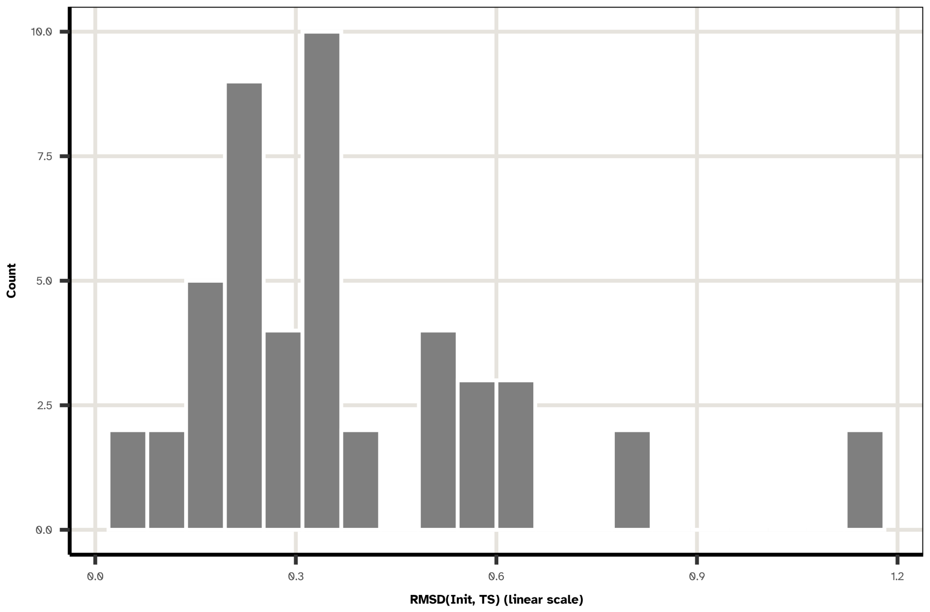


Figure S5: **Input Density.** Histogram of $\text{RMSD}\backslash_{\text{Init}}\backslash_{\text{Final}}$ values, confirming sufficient data density to support the spline term in the regression model.

## S2.7 Model Diagnostics

We performed extensive posterior predictive checks (PPC) and Leave-One-Out (LOO) cross-validation to validate the model fit.

A posterior predictive check works as follows: once the model has been fitted, we draw parameter values from the posterior distribution and use them to simulate new synthetic datasets of the same size as the original data. Each simulated dataset represents one plausible outcome if the experiment were repeated under the same conditions. We then compare the distribution of these simulated datasets to the distribution of the actually observed data. If the model captures the data-generating process adequately, the simulated datasets should look statistically indistinguishable from the real data – their density, spread, and tail behavior should overlap. Systematic discrepancies (e.g., the model consistently predicting too few high-cost outliers) would indicate model misspecification. The figures below show these comparisons.

### S2.7.1 Posterior Predictive Density

Figure S6 compares the observed distribution of gradient calls ($y$, dark line) with 50 distributions simulated from the posterior ($y_{rep}$, light lines). The model accurately reproduces the heavy tail of the cost distribution.

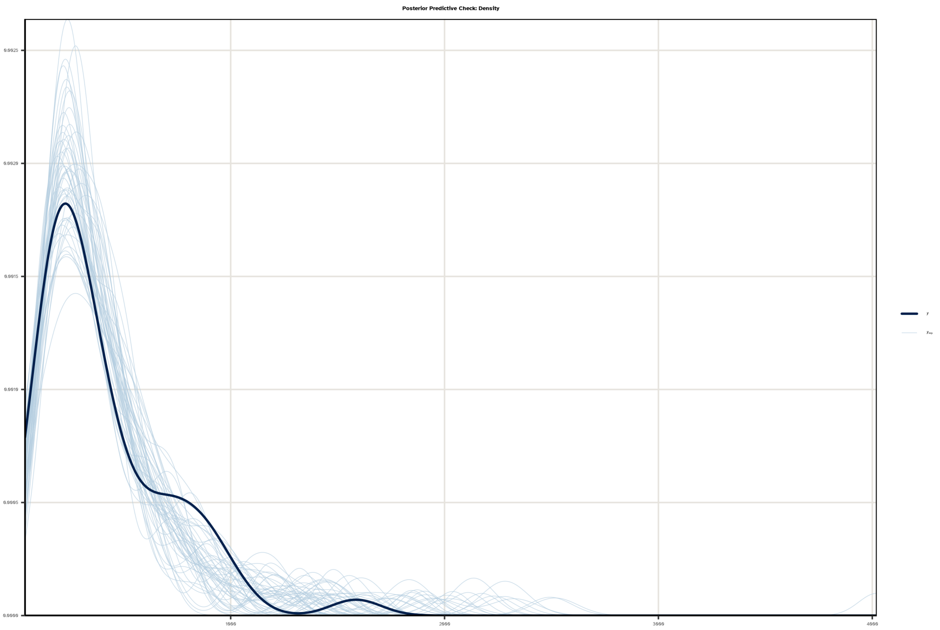


Figure S6: **Posterior predictive density check.** The model successfully captures the data generating process for gradient evaluations.

### S2.7.2 Grouped Intervals

Figure S7 verifies that the model fits both experimental groups (CINEB and OCI-NEB) equally well, with observed data points falling within the predicted credible intervals.

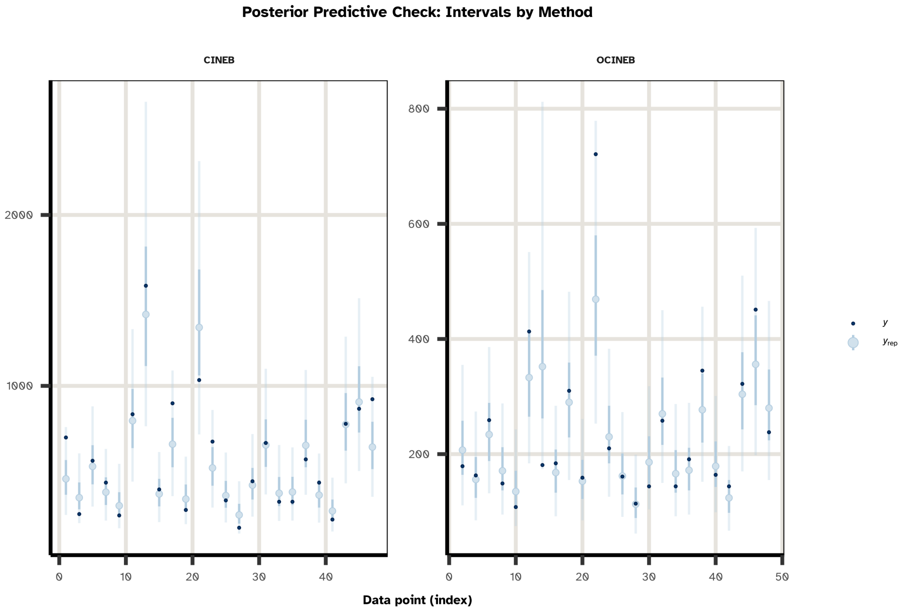


Figure S7: **Posterior predictive intervals grouped by Method.**

### S2.7.3 LOO-PIT and Pareto-k

The Probability Integral Transform (PIT) check (Figure S8) shows that the resulting distribution is approximately uniform, indicating well-calibrated error estimates. The LOO cross-validation identified 7 observations (15%) with Pareto $k > 0.7$, for which moment-matched importance sampling was used to obtain reliable LOO estimates. These high-$k$ observations correspond to systems with extreme speedup ratios (e.g., $HCN+H_2$ at 8.76x), where the model's predictive distribution is most sensitive to individual data points.

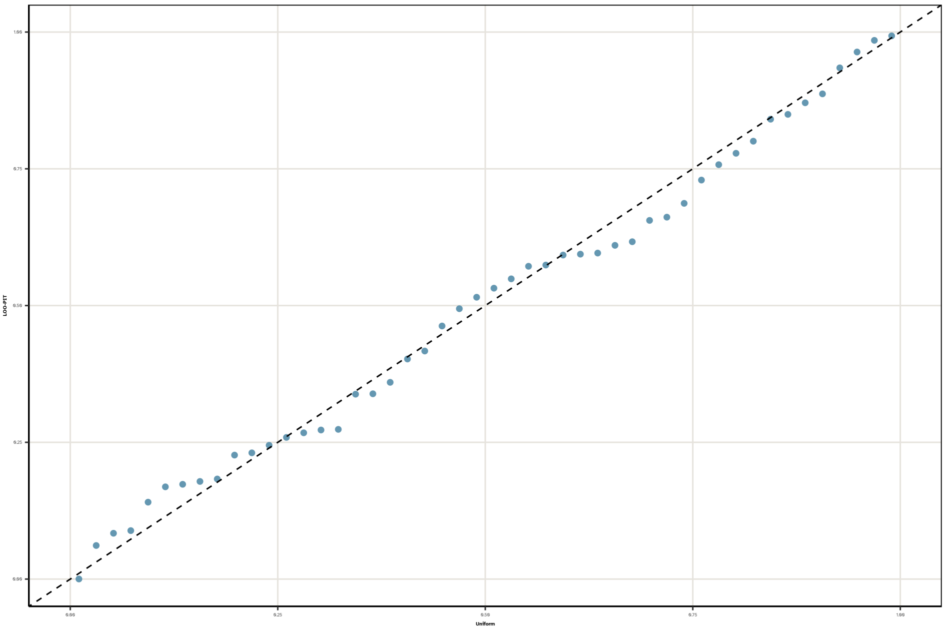

Figure S8: **LOO-PIT Q-Q plot against a uniform distribution.** The alignment with the diagonal indicates the model is well-calibrated.

## S2.8 2D Reaction Landscapes

Figures S9 through S32 present the 2D RMSD-projected reaction landscapes[4] for all 24 Baker-Chan systems. Each pair shows the CI-NEB landscape (left) and OCI-NEB landscape (right). The background contour is a derivative Gaussian process interpolation using an inverse multiquartic kernel. Black dots mark all sampled images; colored circles indicate the final converged path. The white square denotes the saddle point found by each method; the blue star shows the CI-NEB saddle on the OCI-NEB landscape for comparison.

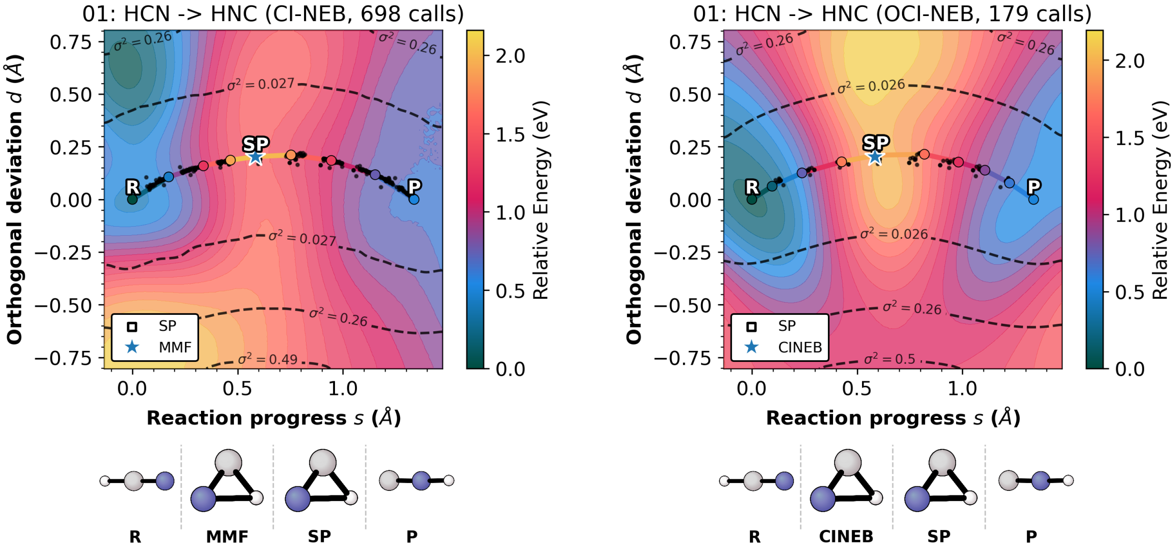


CI-NEB OCI-NEB

Figure S9: HCN $\longrightarrow$ HNC (System 01).

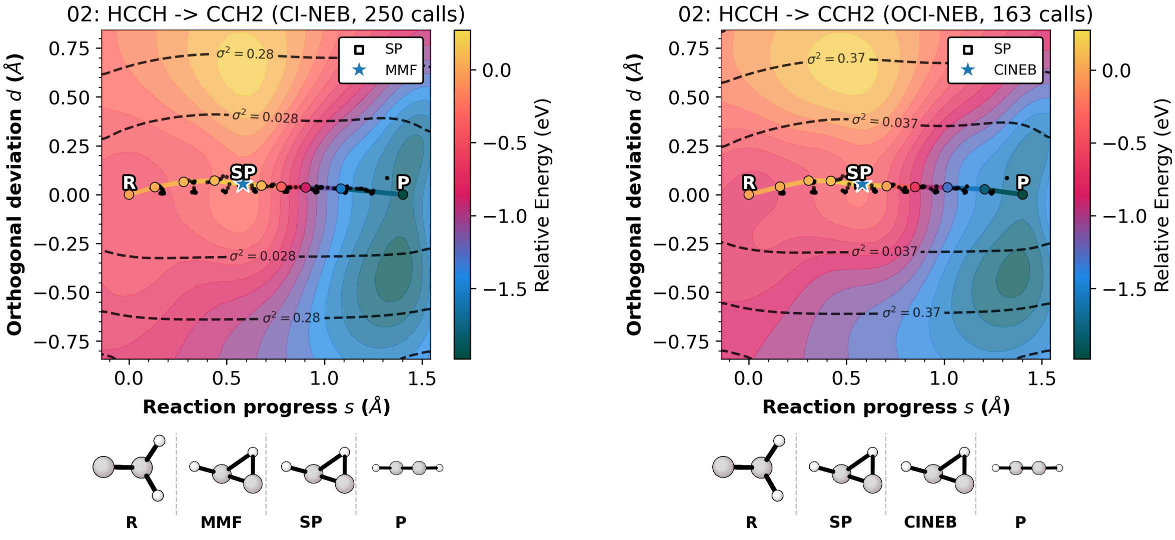


CI-NEB OCI-NEB

Figure S10: HCCH $\longrightarrow$ $CCH_2$ (System 02).

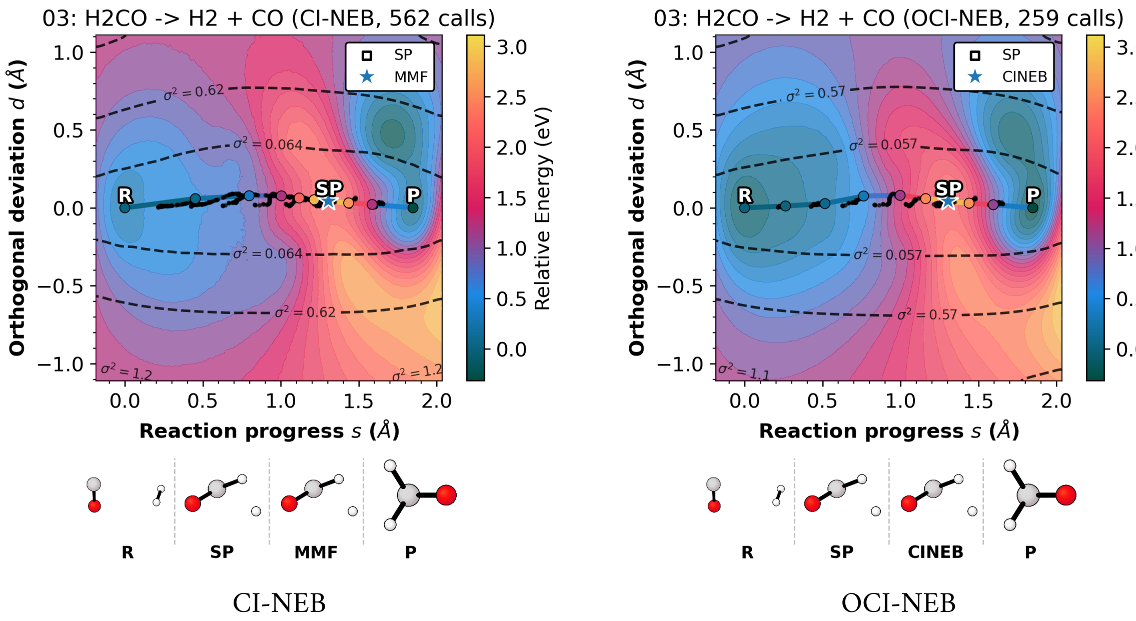


Figure S11: $H_2CO \longrightarrow H_2 + CO$ (System 03).

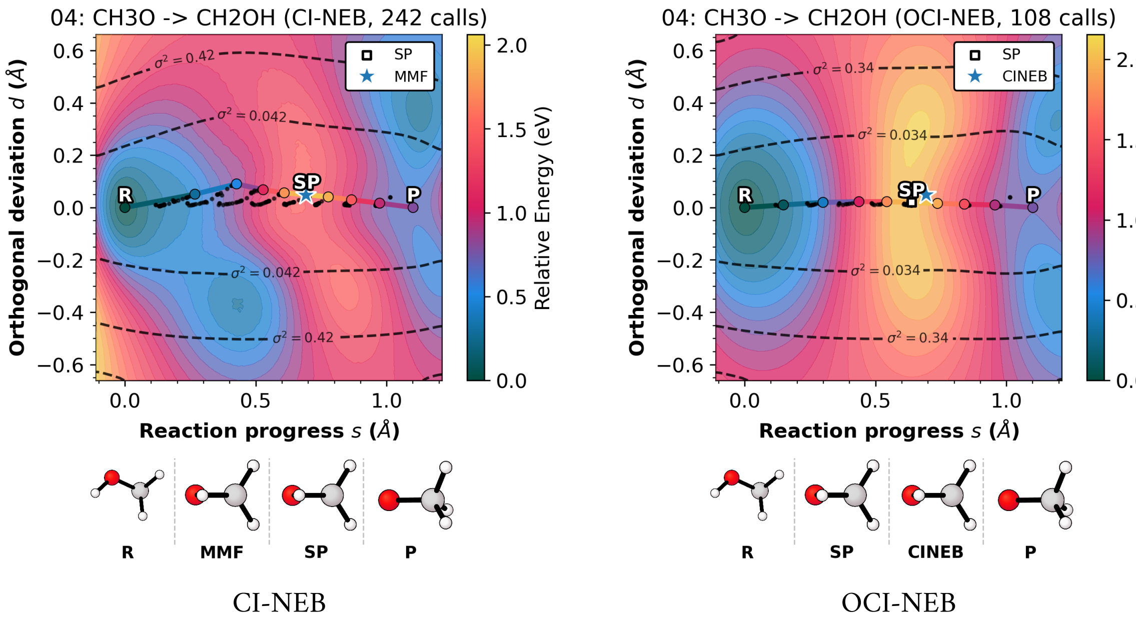


Figure S12: $CH_3O \longrightarrow CH_2OH$ (System 04).

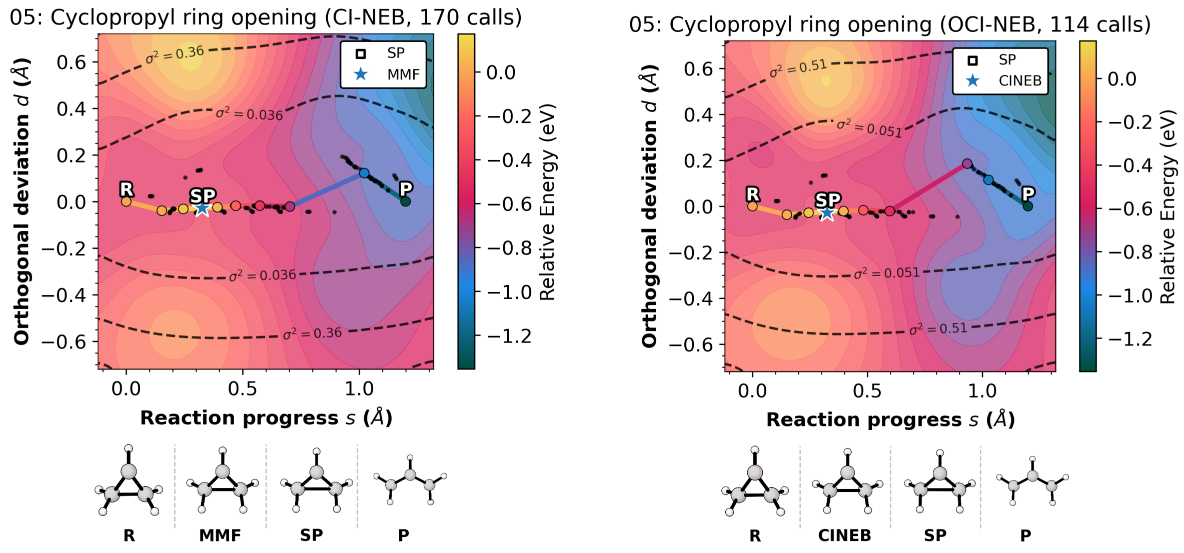


CI-NEB OCI-NEB

Figure S13: Cyclopropyl ring opening (System 05).

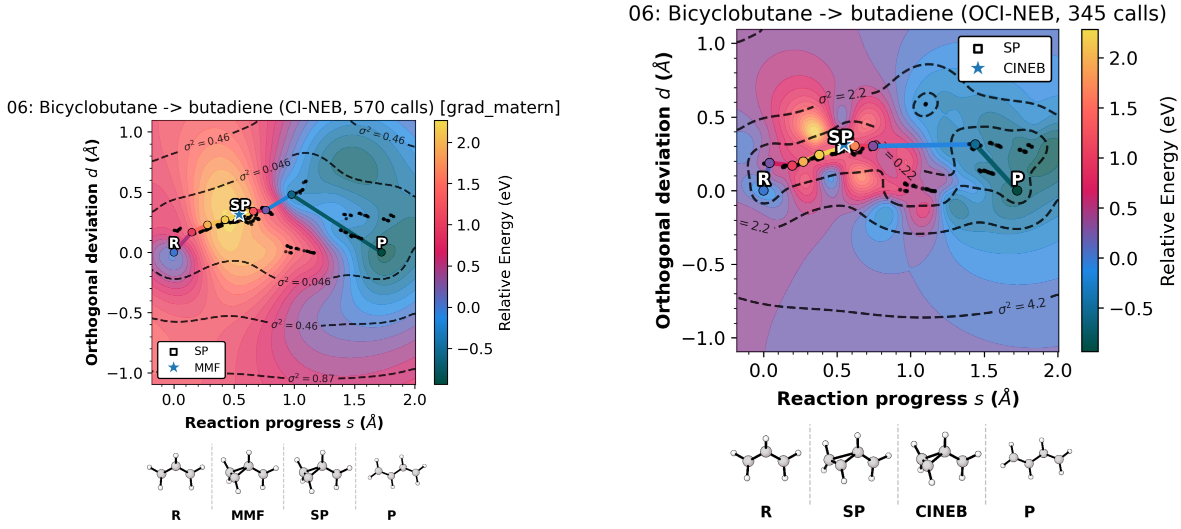


CI-NEB OCI-NEB

Figure S14: Bicyclo[1.1.0]butane $\longrightarrow$ *trans*-butadiene (System 06).

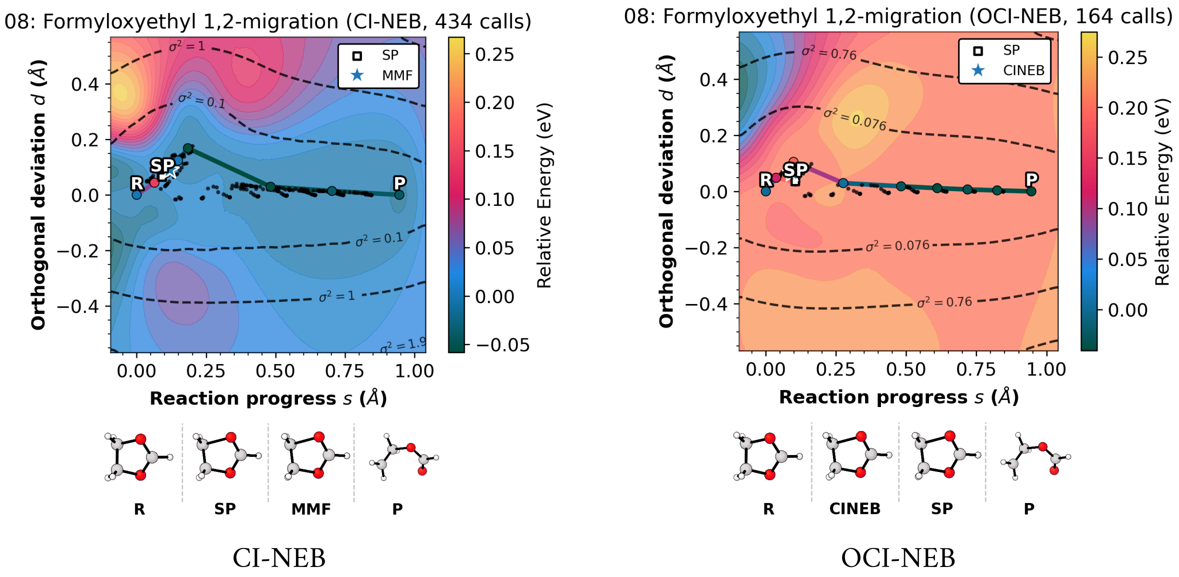


Figure S15: Formyloxyethyl 1,2-migration (System 08).

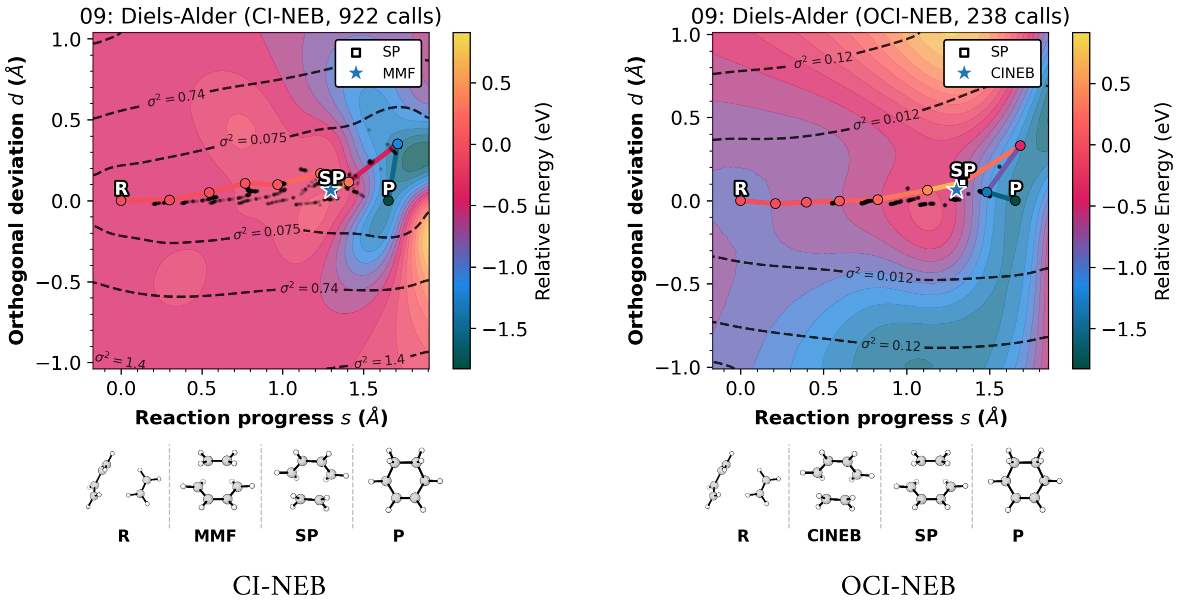


Figure S16: Parent Diels–Alder cycloaddition (System 09).

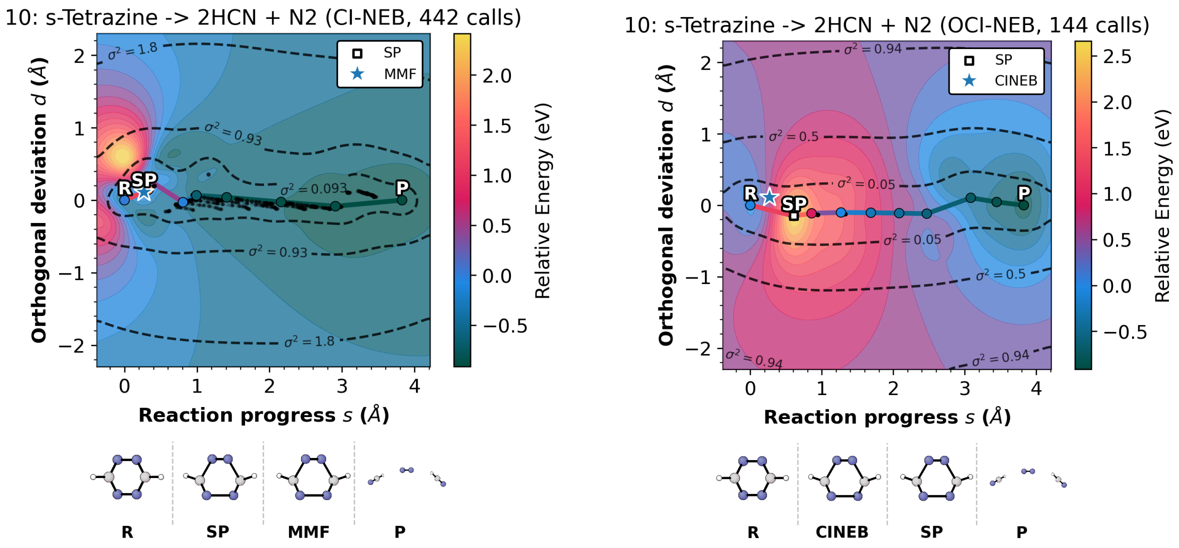


CI-NEB OCI-NEB

Figure S17: *s*-Tetrazine → 2 HCN + $N_2$ (System 10).

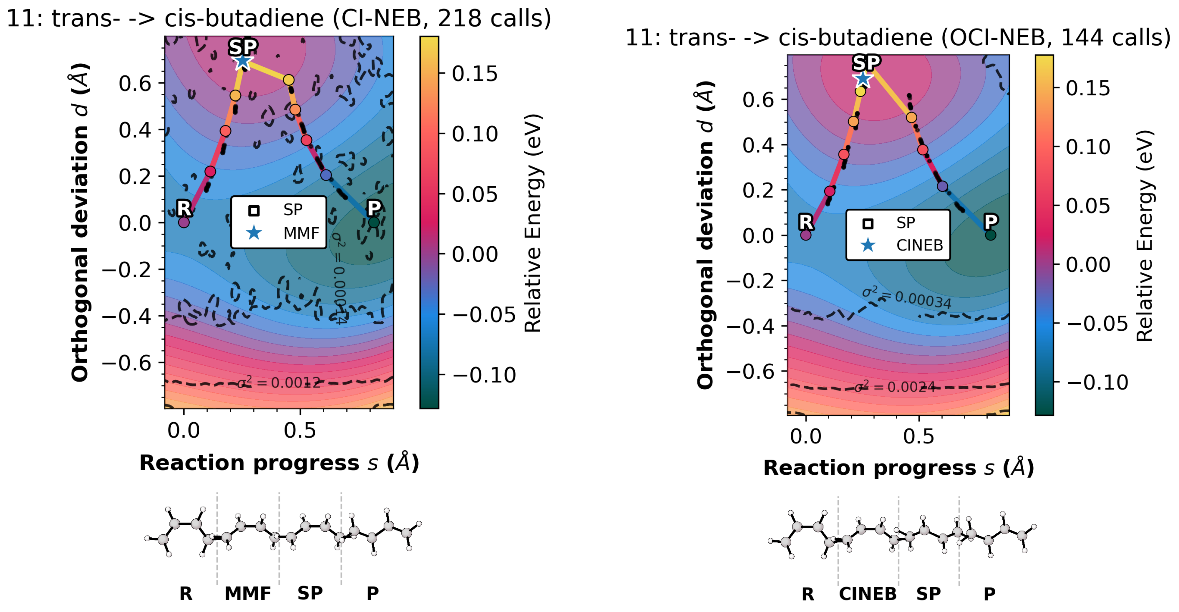


CI-NEB OCI-NEB

Figure S18: *trans*-Butadiene → *cis*-butadiene (System 11).

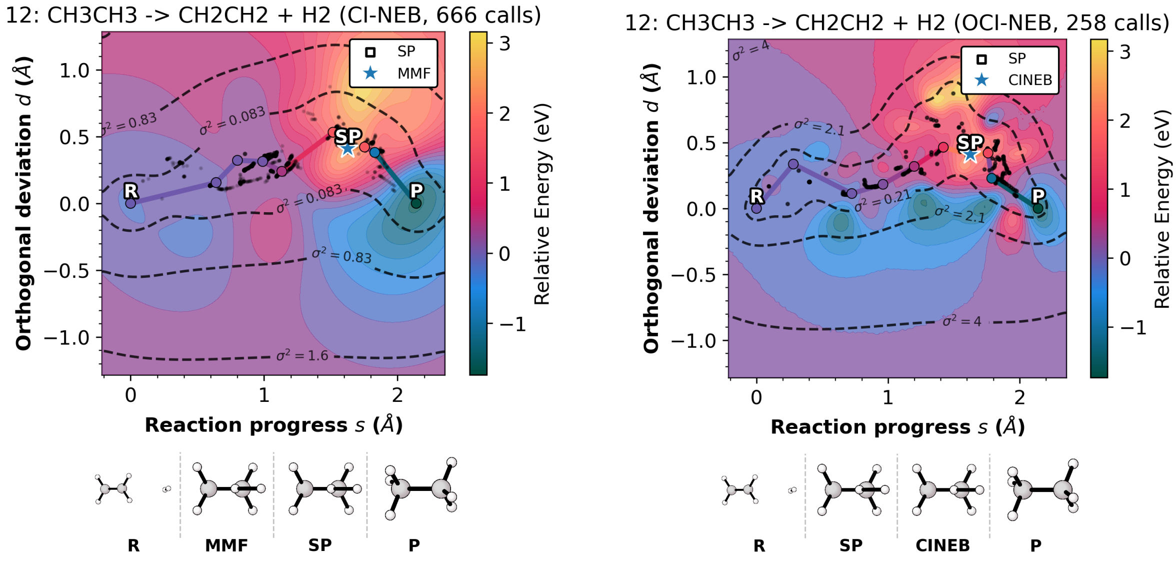


CI-NEB OCI-NEB

Figure S19: $CH_3CH_3 \longrightarrow CH_2CH_2 + H_2$ (System 12).

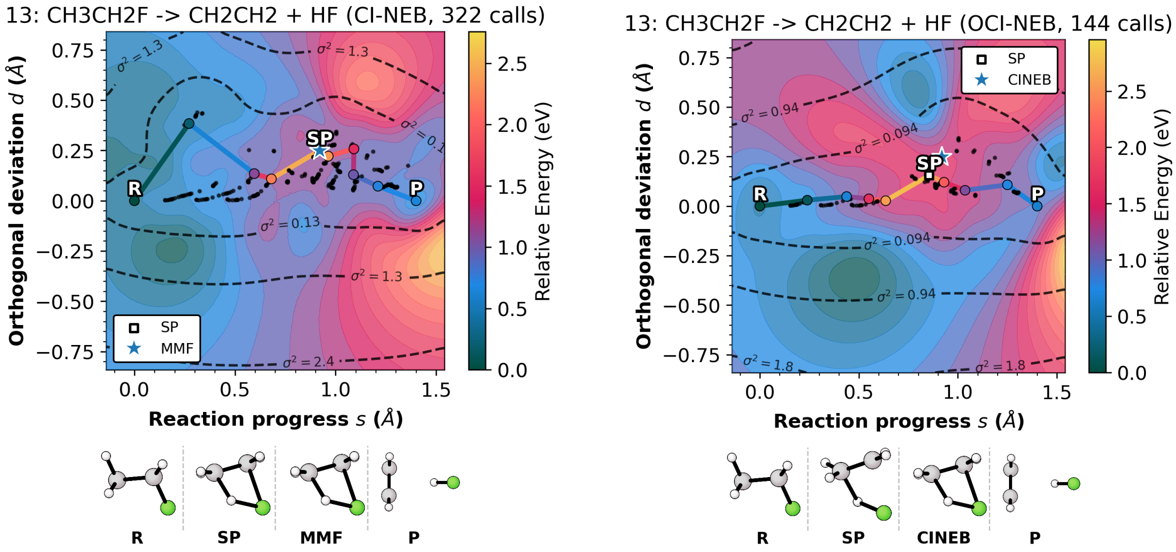


CI-NEB OCI-NEB

Figure S20: $CH_3CH_2F \longrightarrow CH_2CH_2 + HF$ (System 13).

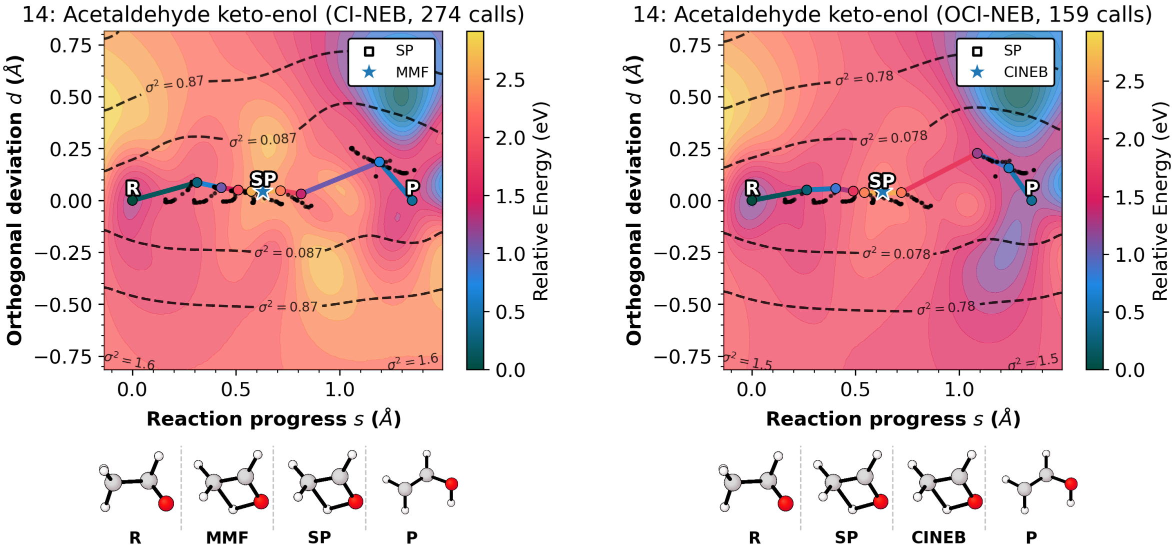


CI-NEB OCI-NEB

Figure S21: Acetaldehyde keto–enol tautomerism (System 14).

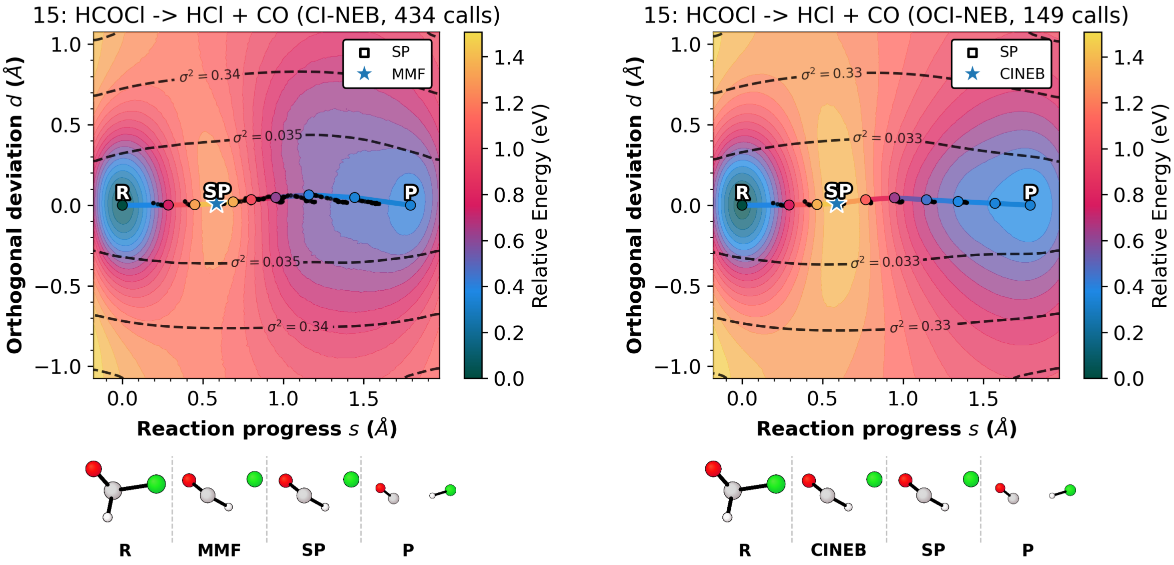


CI-NEB OCI-NEB

Figure S22: HCOCl $\longrightarrow$ HCl + CO (System 15).

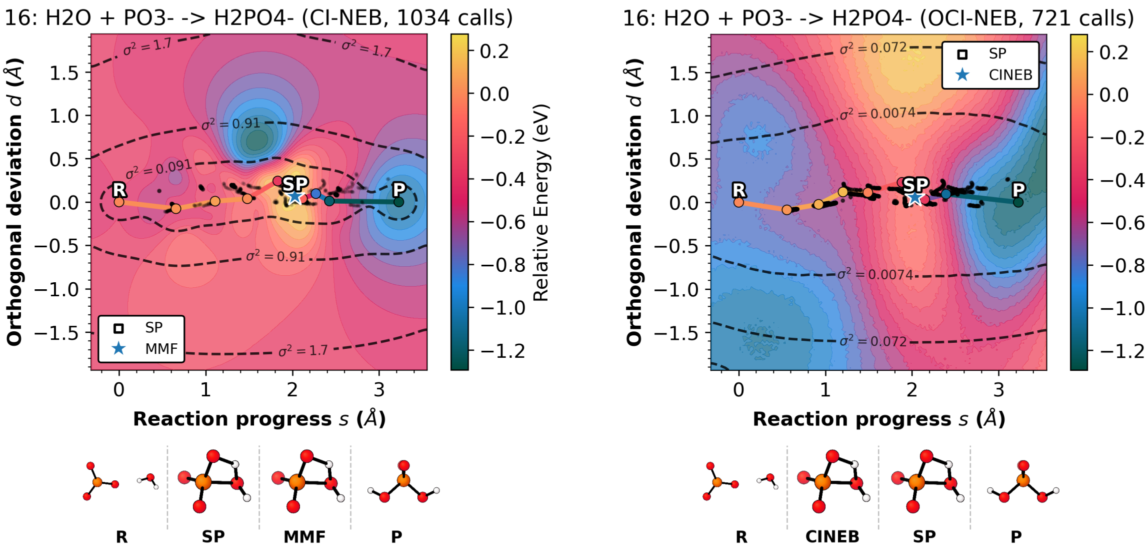


CI-NEB OCI-NEB

Figure S23: $H_2O + PO_3^- \longrightarrow H_2PO_4^-$ (System 16).

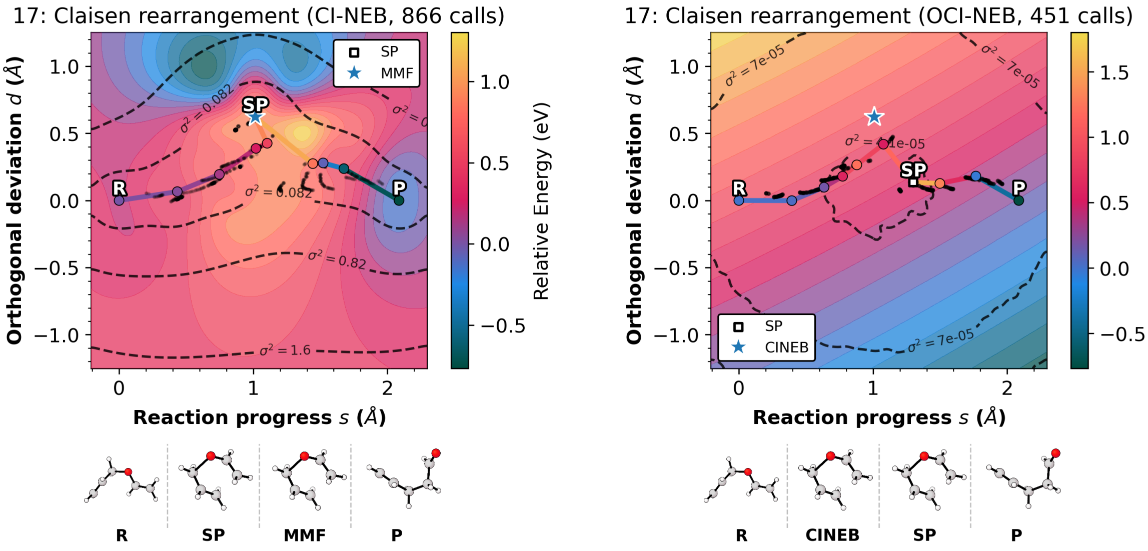


CI-NEB OCI-NEB

Figure S24: $CH_2CHCH_2CH_2CHO$ Claisen rearrangement (System 17).

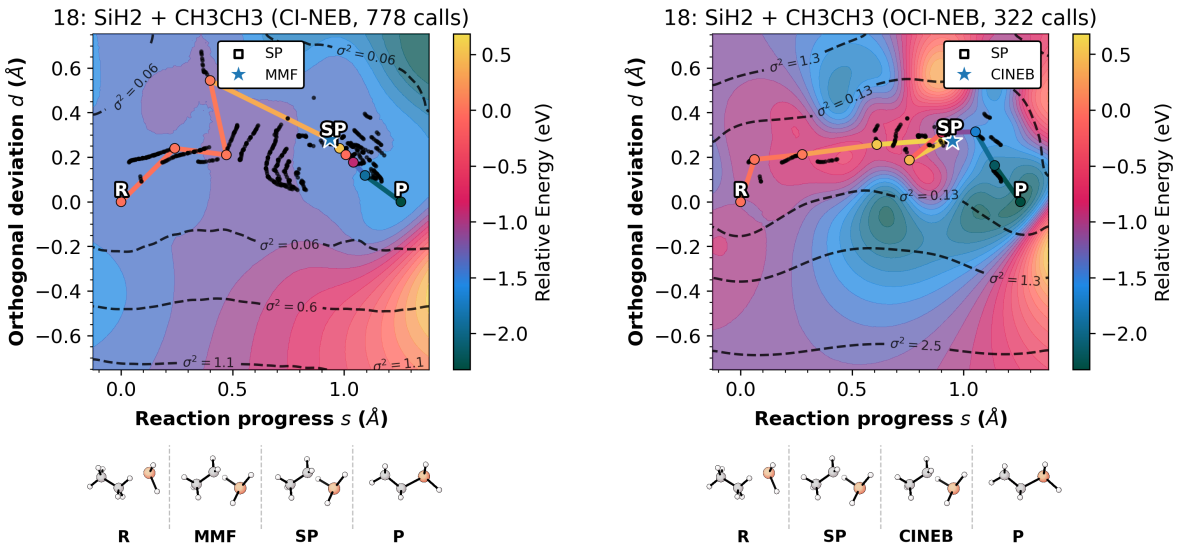


CI-NEB OCI-NEB

Figure S25: $SiH_2 + CH_3CH_3 \longrightarrow SiH_3CH_2CH_3$ (System 18).

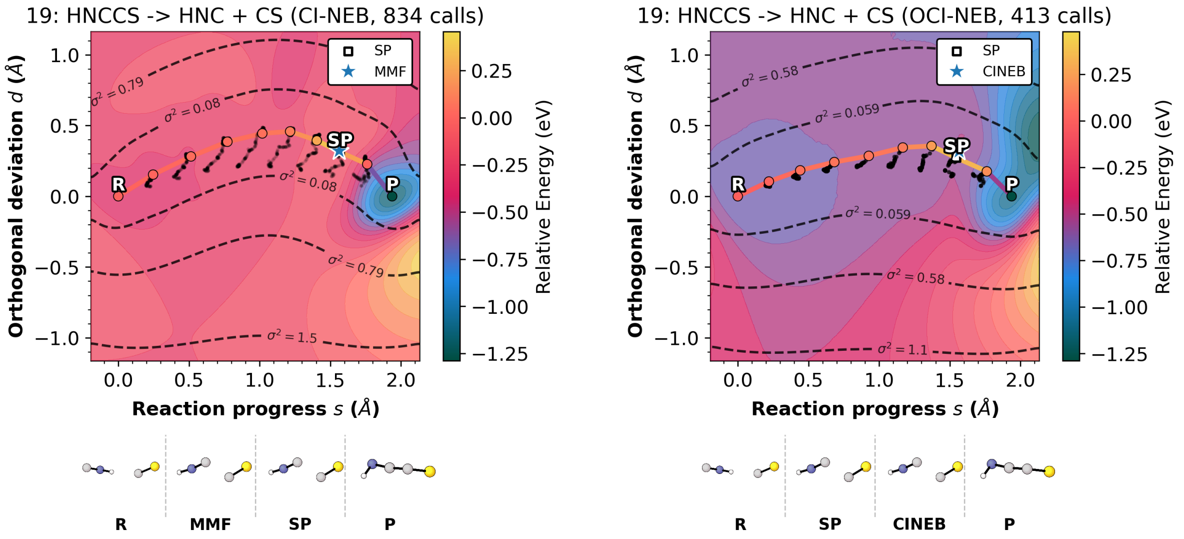


CI-NEB OCI-NEB

Figure S26: HNCCS $\longrightarrow$ HNC + CS (System 19).

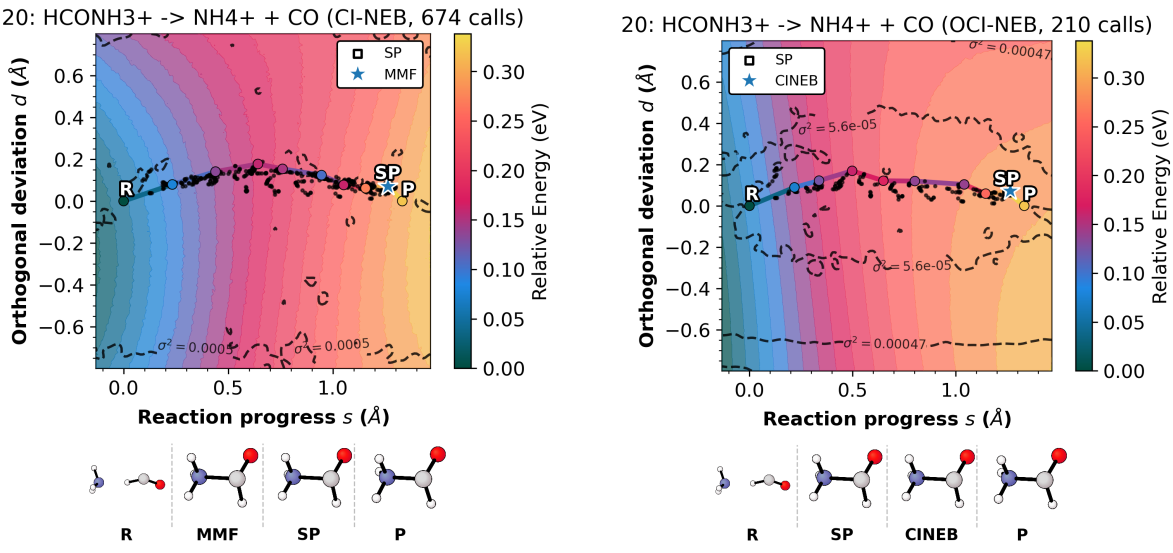


CI-NEB OCI-NEB

Figure S27: $HCONH_3^+ \longrightarrow NH_4^+ + CO$ (System 20).

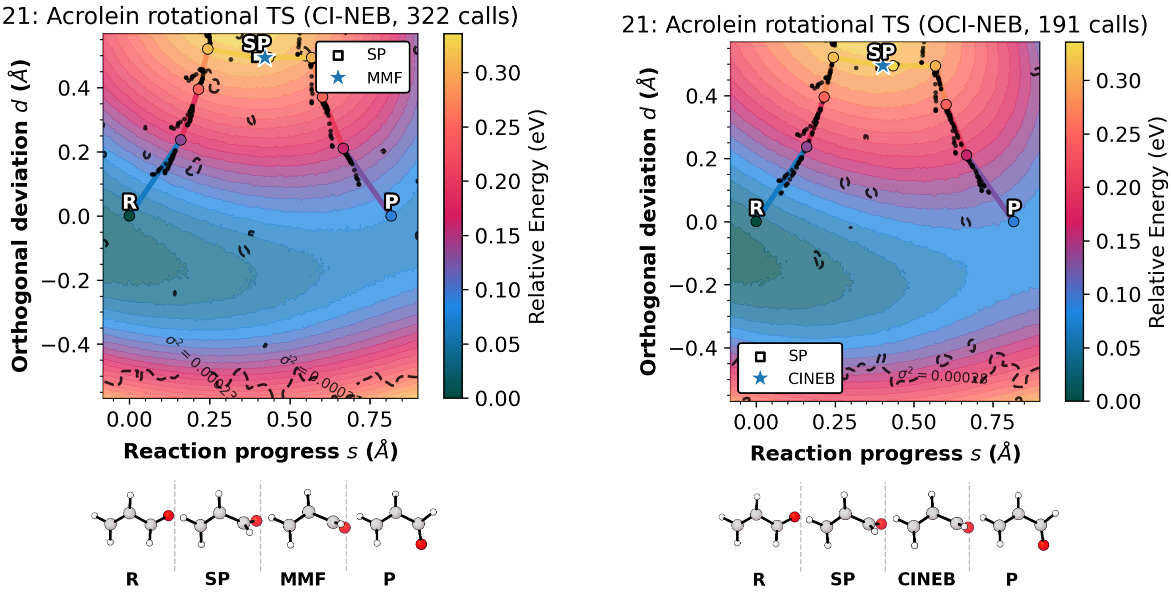


CI-NEB OCI-NEB

Figure S28: Acrolein rotational TS (System 21).

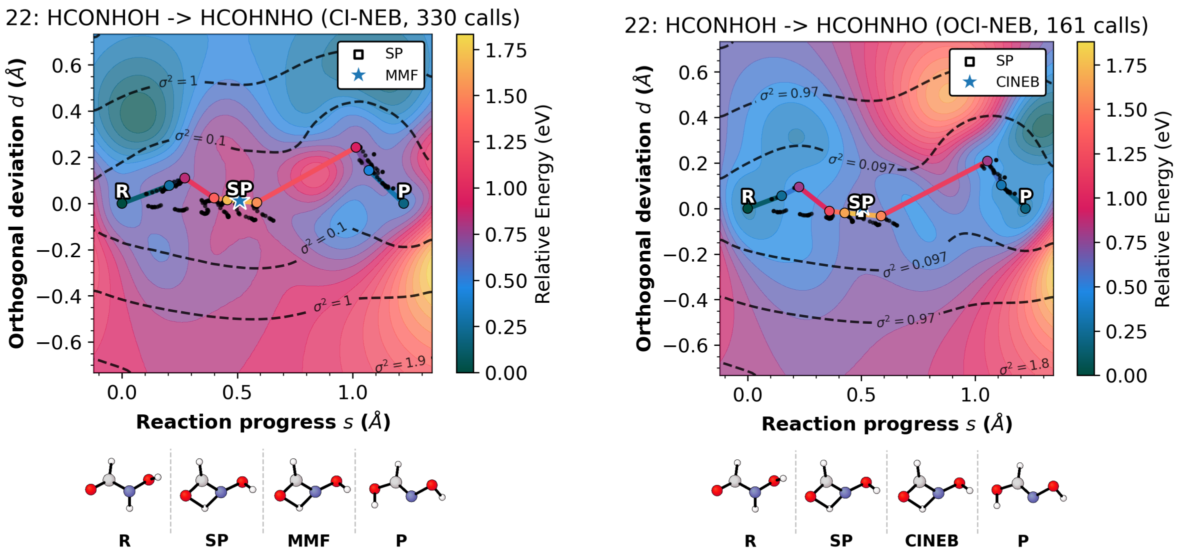


CI-NEB OCI-NEB

Figure S29: HCONHOH ⟶ HCOHNHO (System 22).

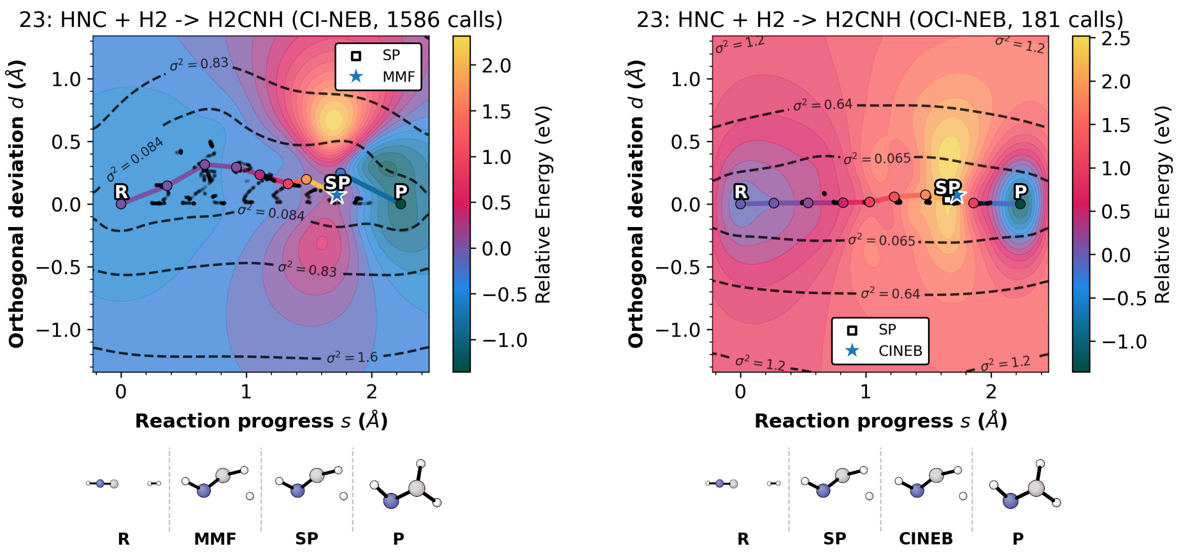


CI-NEB OCI-NEB

Figure S30: HNC + $H_2$ ⟶ $H_2CNH$ (System 23).

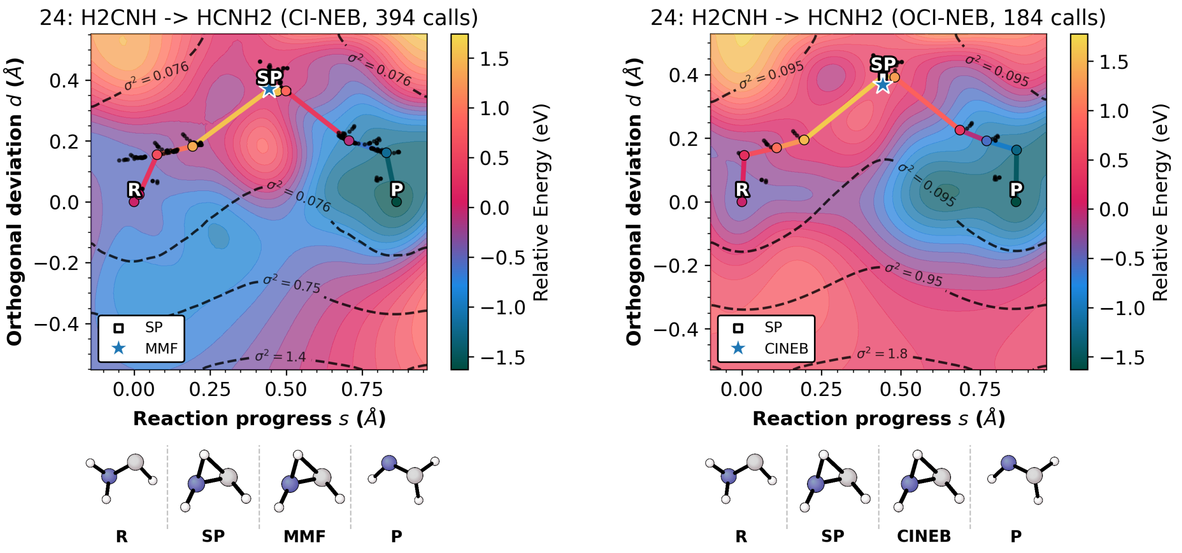


CI-NEB OCI-NEB

Figure S31: $H_2CNH \longrightarrow HCNH_2$ (System 24).

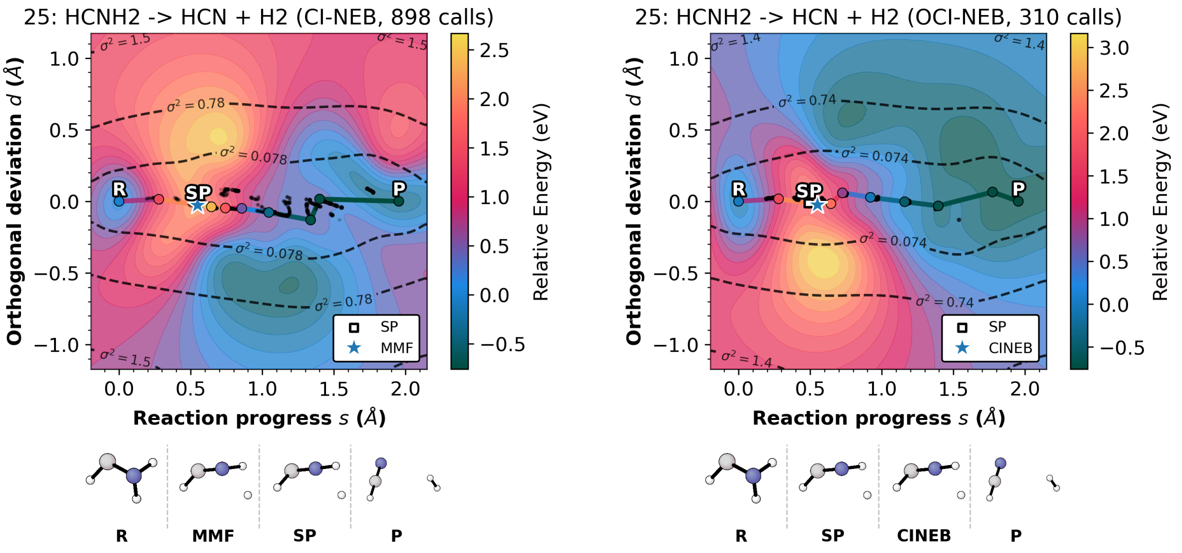


CI-NEB OCI-NEB

Figure S32: $HCNH_2 \longrightarrow HCN + H_2$ (System 25).

## References


[1] Ásgeirsson, V., Birgisson, B. O., Bjornsson, R., Becker, U., Neese, F., Riplinger, C., et al. (2021). Nudged Elastic Band Method for Molecular Reactions Using Energy-Weighted Springs Combined with Eigenvector Following. *Journal of Chemical Theory and Computation* 17, 4929–4945. doi:10.1021/acs.jctc.1c00462

[2] [Dataset] Bigi, F., Abbott, J. W., Loche, P., Mazitov, A., Tisi, D., Langer, M. F., et al. (2025). Metatensor and metatomic: Foundational libraries for interoperable atomistic machine learning. doi:10.48550/arXiv.2508.15704

[3] Goswami, R. (2025). Bayesian hierarchical models for quantitative estimates for performance metrics applied to saddle search algorithms. *AIP Advances* 15, 85210. doi:10.1063/5.0283639

[4] [Dataset] Goswami, R. (2025). Two-dimensional RMSD projections for reaction path visualization and validation. doi:10.48550/arXiv.2512.07329

[5] [Dataset] Mölder, F., Jablonski, K. P., Letcher, B., Hall, M. B., Tomkins-Tinch, C. H., Sochat, V., et al. (2021). Sustainable data analysis with Snakemake. doi:10.12688/f1000research.29032.2
